# Supplementary material for: Swift induction of human spinal lower motor neurons and robust ALS cell screening via single-cell imaging
Source: Stem Cell Reports. 2024 Dec 19;20(1):102377. doi: 10.1016/j.stemcr.2024.11.007 (PMC11784480; doi:10.1016/j.stemcr.2024.11.007)
Supplement: Document S1. Figures S1–S7 and Tables S1–S6 [file mmc1.pdf]

Stem Cell Reports, Volume *20*

Supplemental Information

Swift induction of human spinal lower motor neurons and robust ALS cell screening via single-cell imaging

Selena Setsu, Satoru Morimoto, Shiho Nakamura, Fumiko Ozawa, Kagistia Hana Utami, Ayumi Nishiyama, Naoki Suzuki, Masashi Aoki, Yukio Takeshita, Yukihide Tomari, and Hideyuki Okano

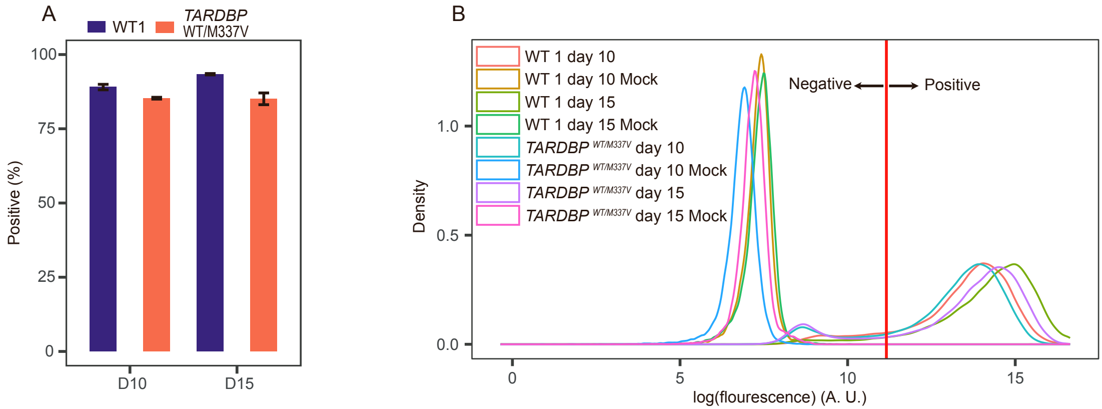

Figure S1 High induction efficiency was confirmed by FACS analysis using Hb9 promoter::mGreenLantern lentivirus

(A) Result of three technical replicates. (B) Gating strategy for negative and positive cells. For mock condition, n = 1. For the other conditions three replicates were combined in this figure.

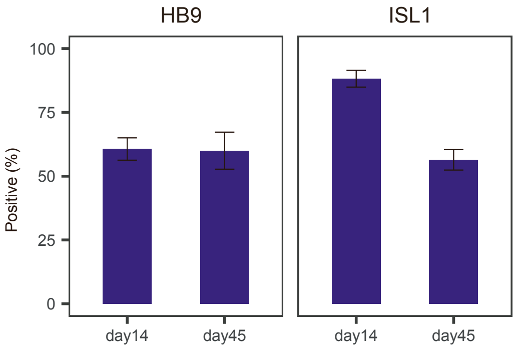

Figure S2 Differentiation efficiency at day 14 and day 45

The error bars are standard deviation. n = 8 biological replicates (different cell lines).

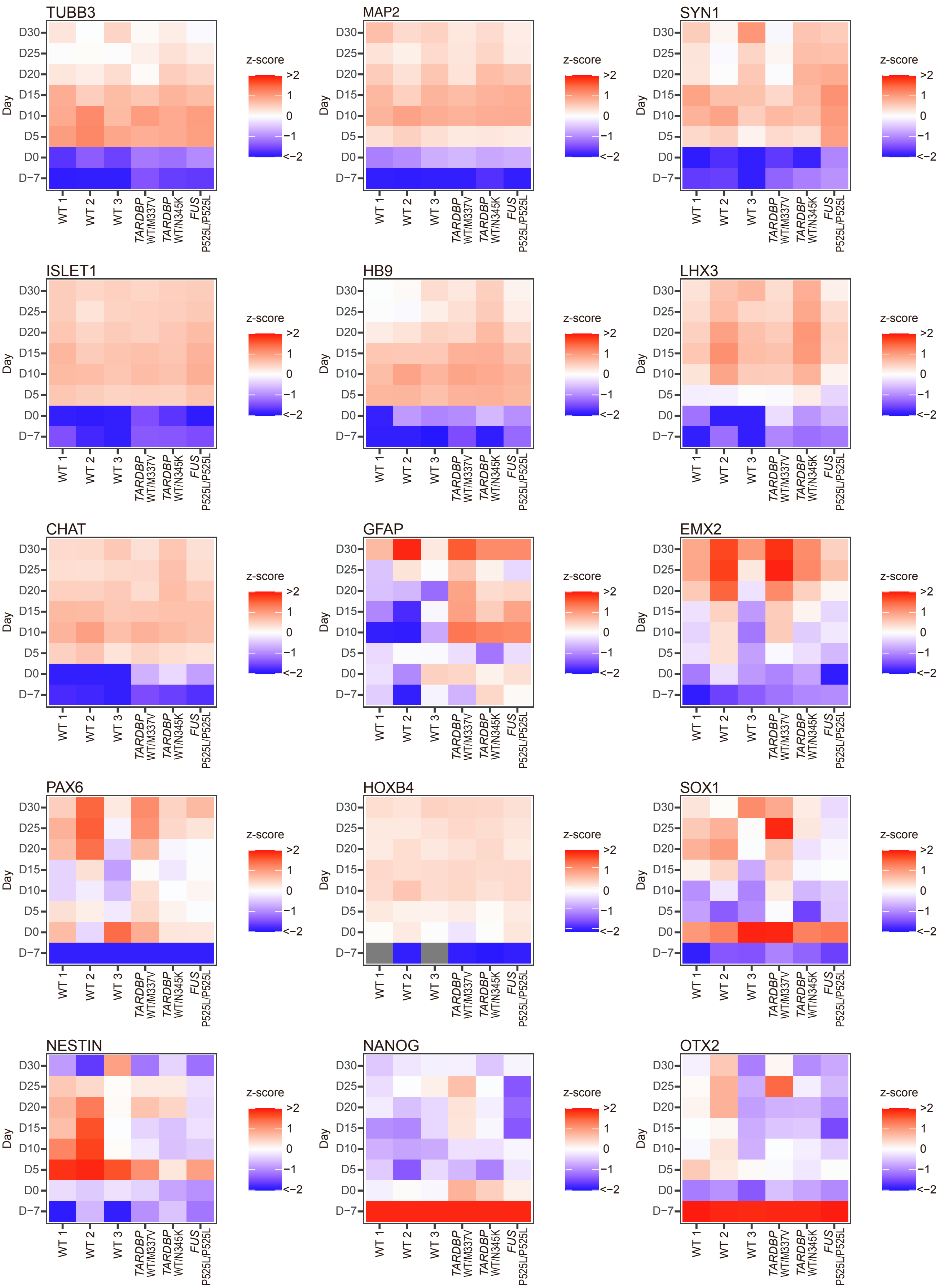

Figure S3 qPCR confirmed LMN characteristic expression until day 30

The expression of each gene is normalized using its z-score. n = 3 technical replicates. Grey represents missing data.

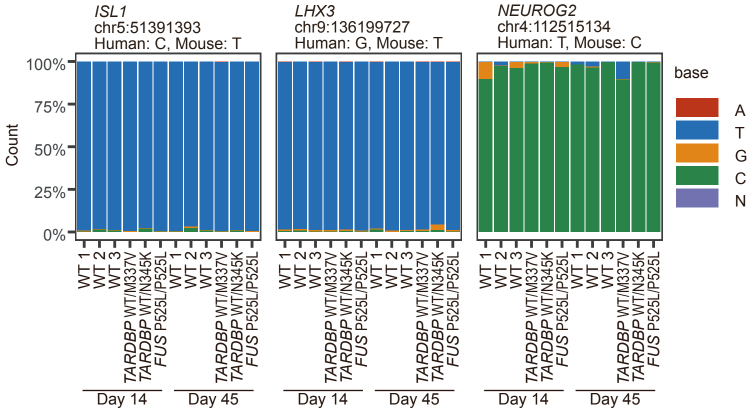

Figure S4 ISL1, LHX3 and NEUROG2 are mostly exogenously expressed in the induced LMNs.

Composition of bases at a location where the sequence of mouse and human are different.

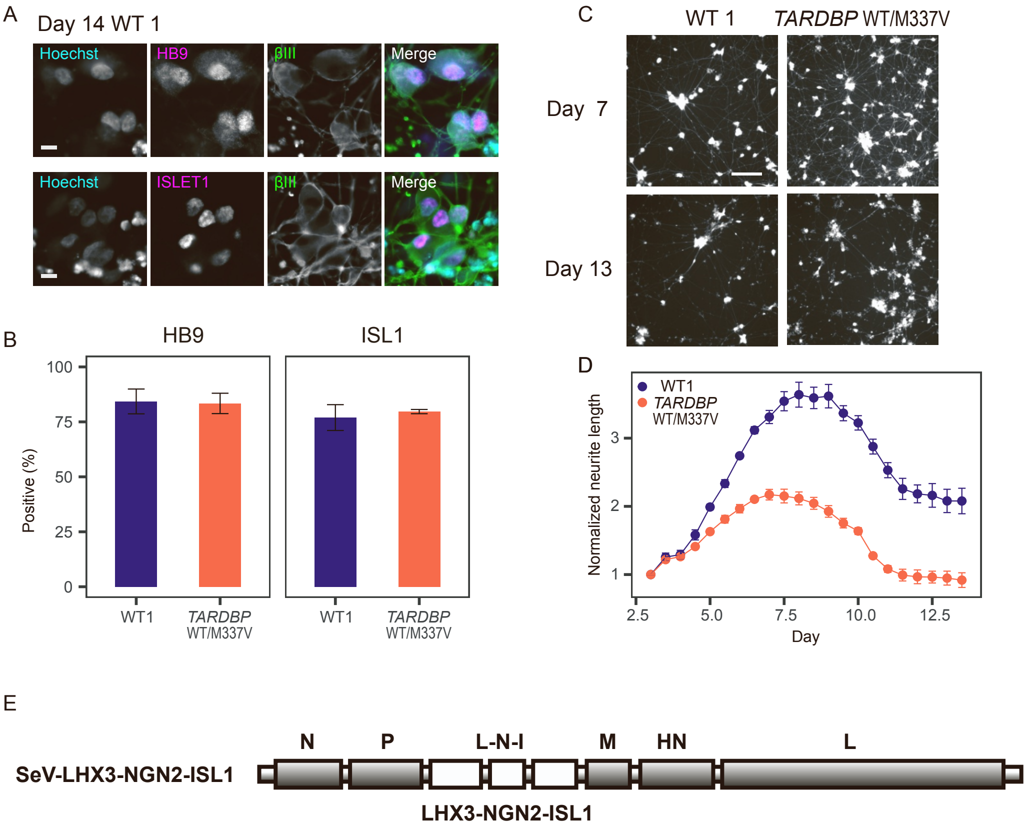

Figure S5 LMN induction using human transcription factors

(A) Representative images. (B) differentiation efficiency at day 14. The error bars are the standard deviation. n = 3 technical replicates. (C) mGreenLantern images of LMNs. (D) Total neurite length of each well was normalized by dividing by corresponding neurite length at day 3. n = 3 technical replicates. (E) Structure of the Sendai virus vector: gray boxes indicate Sendai virus genes, while white boxes represent inserted transcription factors.

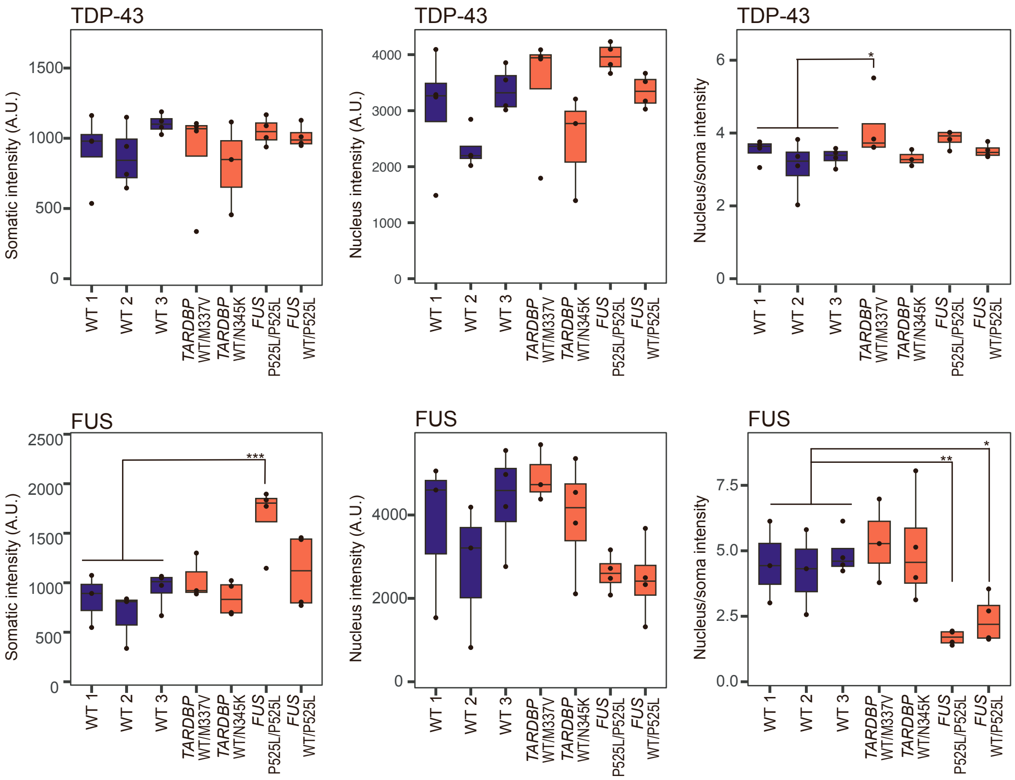

Figure S6 Depletion of FUS proteins from the nucleus in FUS mutant LMNs.

Left panel: Mean intensity of the somatic segment in each cell. Middle panel: Mean intensity of the nucleus segment in each cell. Right panel: Ratio of the mean intensity in the nucleus to mean intensity in the somatic segment of each cell. Results from three to four independent experiments were obtained, and a two-tailed Dunnett's test was performed. All tests were conducted against control cell lines. Unlabeled differences are not significant. *p < 0.05, **p < 0.01; ***p < 0.001.

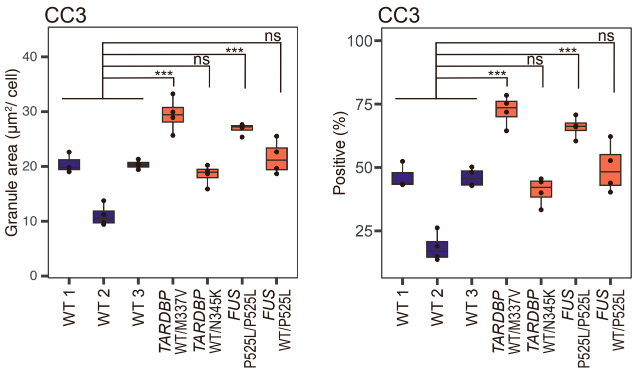

Figure S7 CC3 staining shows increased signal in ALS cell lines

Results from three to four independent experiments were obtained, and a two-tailed Dunnett's test was performed. *p < 0.05; **p < 0.01; ***p < 0.001; ns, non-significant.

Table S1

Number of Analyzed Neurons	23

Axon Metrics	Mean
Neuron Conduction Velocity [m/s]	0.539
Total Axon Length [μm]	2087.665
Longest Branch Length [μm]	864.265
Longest distance from Initiation Site [μm]	920.725
Longest Latency [ms]	2.668
Amplitude at Initiation Site [μV]	116.93

Table S2

Metric	Mean	P90
Firing Rate [Hz]	1.06	2.56
Spike Amplitude [μV]	35.97	52.34
Inter-Spike Interval [ms]	91.73	150.68

Table S3

Name	In the paper	Genotype	Age	Sex	Race	RIKEN BRC ID	Reprogramming methodology	Passage number	Reference
201B7	WT 1	wild type	36	female	Caucasian	HPS0063	Fibroblast, Retrovirus	< 35	Takahashi et al., 2007
1210B2	-	wild type	29	female	Caucasian	HPS4293	Peripheral Blood Mononuclear Cells, Episomal	< 35	Sugai et al., 2016
WD39	WT 2	wild type	16	female	Japanese	NA	Fibroblast, Retrovirus	< 35	Imaizumi, Y. et al.
414C2	WT 3	wild type	36	female	Caucasian	HPS4292	Fibroblast, Episomal	< 35	Okita et al., 2011
A3411	TARDBP WT/M337V	*TARDBP* WT/M337V	62	female	Japanese	HPS0292	Fibroblast, Episomal	< 35	Egawa et al., 2012
SM4-4-5	TARDBP WT/N345K	*TARDBP* WT/N345K*	63	male	Japanese	NA	T cells, Episomal	< 35	Leventoux et al., 2020
FUS-008-1-E6	FUS P525L/P525L	*FUS* P525L/P525L (edited from 201B7)	36	female	Caucasian	NA	NA	< 35	This paper
FUS-008-1-G2	FUS WT/P525L	*FUS* WT/P525L (edited from 201B7)	36	female	Caucasian	NA	NA	< 35	This paper
SM30 No.4	SOD1 ALS	*SOD1* WT/L39R	45	male	Japanese	NA	T cells, Episomal	< 35	This paper
30KALS-11	sporadic ALS	NA	70	male	Japanese	NA	T cells, Episomal	< 35	Morimoto et al., 2023
C9orf72 148-1 No.3	C9orf72 ALS 1	*C90rf72* repeat expansion	63	male	Japanese	NA	T cells, Episomal	< 35	Li et al., 2023
C9orf72 148-4 No.2	C9orf72 ALS 2	*C90rf72* repeat expansion	63	female	Japanese	NA	T cells, Episomal	< 35	This paper

*Medical conditon of SM4-4-5

**Family history**	three cousins with ALS
**Onset of disease**	at the age of 61
**First symptom**	clumsiness in the right hand
**Dementia**	No
**Death**	at the age of 64 due to pneumonia

*Medical conditon of C9orf72 148-1

**Family history**	one sister and one aunt with ALS
**Onset of disease**	at the age of 58
**First symptom**	muscle weakness in the left leg
**Dementia**	No

*Medical conditon of C9orf72 148-4

**Family history**	one brother and one aunt with ALS
**Onset of disease**	at the age of 57
**First symptom**	muscle weakness in the right leg
**Dementia**	No

Table S4

**Target**	**Forward**	**Reverse**
ACTB	TGAAGTGTGACGTGGACATC	GGAGGAGCAATGATCTTGAT
HB9	GTCCACCGCGGGCATGATCC	TCTTCACCTGGGTCTCGGTGAGC
ISLET1	AGCAGCCCAATGACAAAACT	CTGAAAAATTGACCAGTTGCTG
CHAT	GGAGGCGTGGAGCTCAGCGACACC	CGGGGAGCTCGCTGACGGAGTCTG
TUBB3	ATTTCATCTTTGGTCAGAGTGGGGC	TGCAGGCAGTCGCAGTTTTCAC
MAP2	GGCCCAAGCTAAAGTTGGTTCTC	GCAGTGACATCCTCAGCCAAAG
Nanog	TGAACCTCAGCTACAAACAG	TGGTGGTAGGAAGAGTAAAG
Nestin	TTCCCTCAGCTTTCAGGACCCCAA	AAGGCTGGCACAGGTGTCTCAA
GFAP	TGTGAGGCAGAAGCTCCAGGATGA	AGGGTGGCTTCATCTGCTTCCTGT
HOXB4	ACGTGAGCACGGTAAACCCCAA	ATTCCTTCTCCAGCTCCAAGACCT
LHX3	TGCAGGTTTGGTTCCAGAACCGCC	GCCAGGCCTCCATGCTCCAGGGAG
OTX2	ACAAGTGGCCAA TTCACTCC	GAGGTGGACAAGGGA TCTGA
EMX2	GCTTCTAAGGCTGGAACACG	CCAGCTTCTGCCTTTTGAAC
PAX6	ACCACACCGGTTTCCTCCTTCACA	TTGCCATGGTGAAGCTGGGCAT
SOX1	GATCAGCAAGCGCCTGGGGG	AGCAGCGTCTTGGTCTTGCGG
SYN1	ACTTCTGTGACAAGCCCTGG	ACCACGGGGTACGTTGTACT

Table S5

Name	Item number	Host	Isotype	Firm	Dilution
TDP-43 Polyclonal antibody	10782-2-AP	Rabbit	IgG	Proteintech	1:500
FUS Polyclonal Antibody	A300-293A	Rabbit	IgG	Bethyl Laboratories	1:1000
MNR2/HB9/Mnx1 Monoclonal antibody	81.5C10-C	Mouse	IgG1	DSHB	1:150
anti-isl1	39.4D5-C	Mouse	IgG2b	DSHB	1:1000
Purified anti-Tubulin β 3 (TUBB3) Antibody	801202	Mouse	IgG2a	BioLegend	1:2000
Cleaved Caspase-3 (Asp175) Antibody	9661	Rabbit	IgG	Cell Signaling	1:500
Goat anti-Rabbit IgG (H+L) Highly Cross-Adsorbed Secondary Antibody, Alexa Fluor™ 555	A21429	Goat	anti Rabbit IgG	Invitrogen	1:2000
Goat anti-Mouse IgG1 Cross-Adsorbed Secondary Antibody, Alexa Fluor™ 488	A21121	Goat	anti Mouse IgG1	Invitrogen	1:2000
Goat anti-Mouse IgG2a Cross-Adsorbed Secondary Antibody, Alexa Fluor™ 647	A21241	Goat	anti Mouse IgG2a	Invitrogen	1:2000
Goat anti-Mouse IgG2b Cross-Adsorbed Secondary Antibody, Alexa Fluor™ 488	A21141	Goat	anti Mouse IgG2b	Invitrogen	1:2000

Table S6

cell line	target	Class Name	cell count
201B7	HB9	Live_Negative	6665
201B7	HB9	Live_Positive	11269
1210B2	HB9	Live_Negative	5465
1210B2	HB9	Live_Positive	8569
WD39	HB9	Live_Negative	3920
WD39	HB9	Live_Positive	16623
414C2	HB9	Live_Negative	7141
414C2	HB9	Live_Positive	11936
A3411	HB9	Live_Negative	1989
A3411	HB9	Live_Positive	1657
FUS-E6	HB9	Live_Negative	3168
FUS-E6	HB9	Live_Positive	4947
FUS-G2	HB9	Live_Negative	1199
FUS-G2	HB9	Live_Positive	947
SM4	HB9	Live_Negative	10303
SM4	HB9	Live_Positive	4944
201B7	Islet1	Live_Negative	680
201B7	Islet1	Live_Positive	9567
1210B2	Islet1	Live_Negative	407
1210B2	Islet1	Live_Positive	5986
WD39	Islet1	Live_Negative	830
WD39	Islet1	Live_Positive	11300
414C2	Islet1	Live_Negative	783
414C2	Islet1	Live_Positive	10584
A3411	Islet1	Live_Negative	473
A3411	Islet1	Live_Positive	1306
FUS-E6	Islet1	Live_Negative	1373
FUS-E6	Islet1	Live_Positive	4122
FUS-G2	Islet1	Live_Negative	229
FUS-G2	Islet1	Live_Positive	1074
SM4	Islet1	Live_Negative	842
SM4	Islet1	Live_Positive	8726

Table S6, continued

cell line	target	Class Name	n	cell_count
201B7	FUS	Live_Positive	n1	258
201B7	FUS	Live_Positive	n2	414
201B7	FUS	Live_Positive	n3	285
WD39	FUS	Live_Positive	n1	561
WD39	FUS	Live_Positive	n2	718
WD39	FUS	Live_Positive	n3	1646
414C2	FUS	Live_Positive	n1	20
414C2	FUS	Live_Positive	n2	270
414C2	FUS	Live_Positive	n3	381
414C2	FUS	Live_Positive	n4	310
A3411	FUS	Live_Positive	n1	741
A3411	FUS	Live_Positive	n2	434
A3411	FUS	Live_Positive	n3	309
SM4	FUS	Live_Positive	n1	936
SM4	FUS	Live_Positive	n2	202
SM4	FUS	Live_Positive	n3	113
SM4	FUS	Live_Positive	n4	575
FUS-E6	FUS	Live_Positive	n1	34
FUS-E6	FUS	Live_Positive	n2	576
FUS-E6	FUS	Live_Positive	n3	106
FUS-E6	FUS	Live_Positive	n4	267
FUS-G2	FUS	Live_Positive	n1	86
FUS-G2	FUS	Live_Positive	n2	837
FUS-G2	FUS	Live_Positive	n3	709
FUS-G2	FUS	Live_Positive	n4	309
201B7	TDP	Live_Positive	n1	1312
201B7	TDP	Live_Positive	n2	489
201B7	TDP	Live_Positive	n3	1957
201B7	TDP	Live_Positive	n4	1362
WD39	TDP	Live_Positive	n1	1779
WD39	TDP	Live_Positive	n2	240
WD39	TDP	Live_Positive	n3	3571
WD39	TDP	Live_Positive	n4	2541
414C2	TDP	Live_Positive	n1	1176
414C2	TDP	Live_Positive	n2	1004
414C2	TDP	Live_Positive	n3	960
414C2	TDP	Live_Positive	n4	1572
A3411	TDP	Live_Positive	n1	507

Table S6, continued

A3411	TDP	Live_Positive	n2	870
A3411	TDP	Live_Positive	n3	651
A3411	TDP	Live_Positive	n4	415
SM4	TDP	Live_Positive	n1	1842
SM4	TDP	Live_Positive	n2	2728
SM4	TDP	Live_Positive	n3	424
FUS-E6	TDP	Live_Positive	n1	732
FUS-E6	TDP	Live_Positive	n2	467
FUS-E6	TDP	Live_Positive	n3	1016
FUS-E6	TDP	Live_Positive	n4	951
FUS-G2	TDP	Live_Positive	n1	1721
FUS-G2	TDP	Live_Positive	n2	977
FUS-G2	TDP	Live_Positive	n3	1256
FUS-G2	TDP	Live_Positive	n4	1015

Table S6, continued

cell line	target	day	Class Name	cell count
201B7	HB9	day14	Live_Negative	1110
201B7	HB9	day14	Live_Positive	340
201B7	HB9	day45	Live_Negative	3204
201B7	HB9	day45	Live_Positive	1353
1210B2	HB9	day14	Live_Negative	1319
1210B2	HB9	day14	Live_Positive	713
1210B2	HB9	day45	Live_Negative	3032
1210B2	HB9	day45	Live_Positive	1584
WD39	HB9	day14	Live_Negative	2258
WD39	HB9	day14	Live_Positive	1574
WD39	HB9	day45	Live_Negative	4070
WD39	HB9	day45	Live_Positive	2069
414C2	HB9	day14	Live_Negative	1926
414C2	HB9	day14	Live_Positive	924
414C2	HB9	day45	Live_Negative	1736
414C2	HB9	day45	Live_Positive	2727
A3411	HB9	day14	Live_Negative	773
A3411	HB9	day14	Live_Positive	738
A3411	HB9	day45	Live_Negative	4942
A3411	HB9	day45	Live_Positive	643
SM4	HB9	day14	Live_Negative	1394
SM4	HB9	day14	Live_Positive	543
SM4	HB9	day45	Live_Negative	5657
SM4	HB9	day45	Live_Positive	1206
FUS-E6	HB9	day14	Live_Negative	534
FUS-E6	HB9	day14	Live_Positive	384
FUS-E6	HB9	day45	Live_Negative	7507
FUS-E6	HB9	day45	Live_Positive	564
FUS-G2	HB9	day14	Live_Negative	1929
FUS-G2	HB9	day14	Live_Positive	535
FUS-G2	HB9	day45	Live_Negative	2507
FUS-G2	HB9	day45	Live_Positive	1736
201B7	ISL1	day14	Live_Negative	593
201B7	ISL1	day14	Live_Positive	2610
201B7	ISL1	day45	Live_Negative	2904
201B7	ISL1	day45	Live_Positive	721
1210B2	ISL1	day14	Live_Negative	315
1210B2	ISL1	day14	Live_Positive	1990

Table S6, continued

1210B2	ISL1	day45	Live_Negative	3599
1210B2	ISL1	day45	Live_Positive	1191
WD39	ISL1	day14	Live_Negative	755
WD39	ISL1	day14	Live_Positive	4601
WD39	ISL1	day45	Live_Negative	4317
WD39	ISL1	day45	Live_Positive	1377
414C2	ISL1	day14	Live_Negative	362
414C2	ISL1	day14	Live_Positive	3692
414C2	ISL1	day45	Live_Negative	2268
414C2	ISL1	day45	Live_Positive	1663
A3411	ISL1	day14	Live_Negative	511
A3411	ISL1	day14	Live_Positive	2707
A3411	ISL1	day45	Live_Negative	5626
A3411	ISL1	day45	Live_Positive	263
SM4	ISL1	day14	Live_Negative	242
SM4	ISL1	day14	Live_Positive	2619
SM4	ISL1	day45	Live_Negative	4434
SM4	ISL1	day45	Live_Positive	1077
FUS-E6	ISL1	day14	Live_Negative	285
FUS-E6	ISL1	day14	Live_Positive	1459
FUS-E6	ISL1	day45	Live_Negative	6170
FUS-E6	ISL1	day45	Live_Positive	411
FUS-G2	ISL1	day14	Live_Negative	629
FUS-G2	ISL1	day14	Live_Positive	3326
FUS-G2	ISL1	day45	Live_Negative	2238
FUS-G2	ISL1	day45	Live_Positive	950

Table S6, continued

cell line	target	Class Name	n	cell_count
201B7	CC3	Live_Positive	n1	1078
201B7	CC3	Live_Positive	n2	1506
201B7	CC3	Live_Positive	n3	789
WD39	CC3	Live_Positive	n1	2065
WD39	CC3	Live_Positive	n2	1435
WD39	CC3	Live_Positive	n3	3544
WD39	CC3	Live_Positive	n4	2974
414C2	CC3	Live_Positive	n1	1288
414C2	CC3	Live_Positive	n2	968
414C2	CC3	Live_Positive	n3	1030
414C2	CC3	Live_Positive	n4	1929
A3411	CC3	Live_Positive	n1	712
A3411	CC3	Live_Positive	n2	824
A3411	CC3	Live_Positive	n3	571
A3411	CC3	Live_Positive	n4	242
SM4	CC3	Live_Positive	n1	1765
SM4	CC3	Live_Positive	n2	928
SM4	CC3	Live_Positive	n3	2427
SM4	CC3	Live_Positive	n4	325
FUS-E6	CC3	Live_Positive	n1	718
FUS-E6	CC3	Live_Positive	n2	687
FUS-E6	CC3	Live_Positive	n3	1023
FUS-E6	CC3	Live_Positive	n4	599
FUS-G2	CC3	Live_Positive	n1	1598
FUS-G2	CC3	Live_Positive	n2	693
FUS-G2	CC3	Live_Positive	n3	1751
FUS-G2	CC3	Live_Positive	n4	994
