# Supplementary material for: Swift induction of human spinal lower motor neurons and robust ALS cell screening via single-cell imaging
Source: Stem Cell Reports. 2024 Dec 19;20(1):102377. doi: 10.1016/j.stemcr.2024.11.007 (PMC11784480; doi:10.1016/j.stemcr.2024.11.007)
Supplement: Document S2. Article plus supplemental information [file mmc3.pdf]

Stem Cell Reports
Resource
OPEN ACCESS

Swift induction of human spinal lower motor neurons and robust ALS cell screening via single-cell imaging

Selena Setsu,[1,2,3] Satoru Morimoto,[1,4,9,*] Shiho Nakamura,[1,4] Fumiko Ozawa,[1,4] Kagistia Hana Utami,[1,4] Ayumi Nishiyama,[5] Naoki Suzuki,[5,6] Masashi Aoki,[5] Yukio Takeshita,[7,8] Yukihide Tomari,[2,3] and Hideyuki Okano[1,4,*]
[1]Keio University Regenerative Medicine Research Center, Kanagawa 210-0821, Japan
[2]Laboratory of RNA Function, Institute for Quantitative Biosciences, The University of Tokyo, Bunkyo-ku, Tokyo 113-0032, Japan
[3]Department of Computational Biology and Medical Sciences, Graduate School of Frontier Sciences, The University of Tokyo, Bunkyo-ku, Tokyo 113-0032, Japan
[4]Division of Neurodegenerative Disease Research, Tokyo Metropolitan Institute for Geriatrics and Gerontology, Tokyo 173-0015, Japan
[5]Department of Neurology, Tohoku University Graduate School of Medicine, Sendai 980-8575, Japan
[6]Department of Rehabilitation Medicine, Tohoku University Graduate School of Medicine, Sendai, Japan
[7]Department of Neurology and Clinical Neuroscience, Yamaguchi University Graduate School of Medicine, Yamaguchi 753-8511, Japan
[8]Department of Neurotherapeutics, Yamaguchi University Graduate School of Medicine, Yamaguchi 753-8511, Japan
[9]Lead contact
*Correspondence: satoru_morimoto@keio.jp (S.M.), hidokano@keio.jp (H.O.)
https://doi.org/10.1016/j.stemcr.2024.11.007

SUMMARY

This study introduces a novel method for rapidly and efficiently inducing human spinal lower motor neurons (LMNs) from induced pluripotent stem cells (iPSCs) to eventually elucidate the pathomechanisms of amyotrophic lateral sclerosis (ALS) and facilitate drug screening. Previous methods were limited by low induction efficiency, poor LMN purity, or labor-intensive induction and evaluation processes. Our protocol overcomes these challenges, achieving around 80% induction efficiency within just two weeks by combining a small molecule-based approach with transcription factor transduction. Moreover, to exclude non-LMN cells from the analysis, we utilized time-lapse microscopy and machine learning to analyze the morphology and viability of iPSC-derived LMNs on a single-cell basis, establishing an effective pathophysiological evaluation system. This rapid, efficient, and streamlined protocol, along with our single-cell-based evaluation method, enables large-scale analysis and drug screening using iPSC-derived motor neurons.

INTRODUCTION

Amyotrophic lateral sclerosis (ALS) is a neurodegenerative disease characterized by late-onset, progressive, and fatal motor neuron (MN) degeneration, leading to muscle weakness, respiratory failure, and ultimately death. Despite intensive research efforts, the underlying pathomechanisms of ALS remain poorly understood, creating a high demand for fast methods to evaluate therapy effectiveness. Induced pluripotent stem cells (iPSCs) have emerged as a powerful tool for drug screening and understanding the mechanisms underlying ALS pathology (Takahashi and Yamanaka, 2006; Takahashi et al., 2007; Sances et al., 2016; Goto et al., 2017; Fujimori et al., 2018; Shin et al., 2018; Ito et al., 2023; Okano and Morimoto, 2022; Okano et al., 2020). However, iPSC-based research still faces significant challenges, including low induction efficiency, poor MN purity, and labor-intensive induction and evaluation procedures (Lamas and Roybon, 2021; Castillo Bautista and Sterneckert, 2023).

These problems have constrained most iPSC-based ALS disease model studies to cells with clear genetic abnormalities and small sample sizes, limiting their applicability to sporadic cases of ALS. Sporadic ALS, which comprises the majority of ALS cases, lacks obvious genetic abnormalities, exhibits high heterogeneity in disease course, and occurs in patients with various backgrounds. Accurate analysis would thus seem to require a large number of iPSCs derived from many sporadic ALS patients. An efficient neural induction method with clear endpoints is essential for utilizing a large iPSC library.

In this study, we developed a novel protocol for the rapid and efficient induction of human spinal lower motor neurons (LMNs) from iPSCs, targeting the spinal cord, a major locus of ALS pathogenesis. Our protocol achieved around 80% induction efficiency within just two weeks, a significant improvement over conventional methods (Sances et al., 2016; Goto et al., 2017). Time-lapse microscopy and machine learning allowed us to analyze the morphology and viability of iPSC-derived neurons at the single-cell level, establishing an effective pathophysiological evaluation system for neurodegenerative diseases. This approach has the potential to serve as a key tool for large-scale analysis and drug screening using iPSCs derived from ALS patients, facilitating the development of ALS therapies.

© 2024 The Author(s). Published by Elsevier Inc. on behalf of International Society for Stem Cell Research.
This is an open access article under the CC BY license (http://creativecommons.org/licenses/by/4.0/).

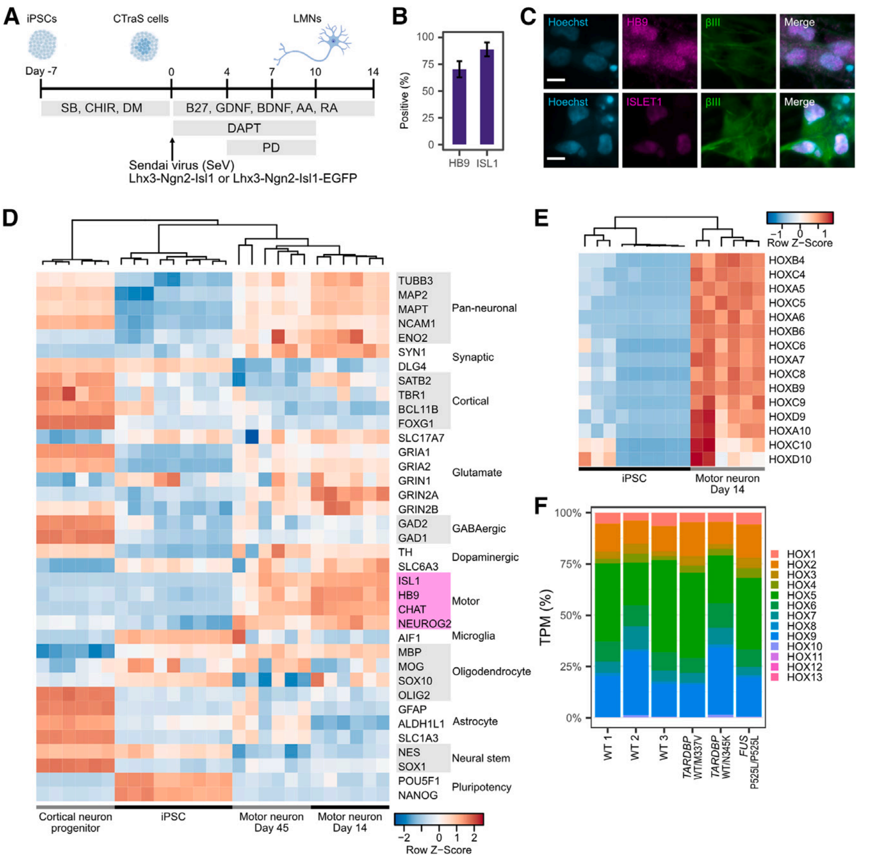

Figure 1. High efficiency and accuracy in differentiating spinal lower motor neurons

(A) Schematic illustration of the differentiation protocol. Sendai viruses were applied at MOI 5.

(B) Differentiation efficiency at day 7 based on immunocytochemistry of HB9 and ISLET. Error bar is the standard deviation. *n* = 8 biological replicates (different cell lines).

(C) Representative immunocytochemistry image of induced LMNs stained for HB9 (magenta), ISLET1 (magenta), and TUBB3 (green), and counterstained with Hoechst (cyan). Day 7 is shown. Scale bar, 10 μm.

(D) Expression profile of major marker genes in accordance with RNA-seq analysis. Data of cortical neuron progenitors and iPSCs were downloaded from the Sequence Read Archive (SRA) dataset. BioProject IDs are PRJNA 660028, PRJNA801842, and PRJNA803470. Cell lines used for MN day 14 and 45 are three WT lines (201B7, WD39, and 414C2) and three ALS lines (A3411 ($TARDBP^{WT/M337V}$), SM4-4-5 ($TARDBP^{WT/N345K}$), and FUS-008-1-E6 ($FUS^{P525L/P525L}$)). Marker genes for motor neuron are highlighted in magenta.

(E) Expression profile of major HOX regional marker genes for spinal cord in accordance with RNA-seq analysis.

(F) Hox gene expression pattern was calculated based on TPM. TPM expression of each group of Hox genes was summed and divided by total Hox gene expression.

RESULTS

Rapid and efficient induction of human spinal LMNs

To achieve rapid and efficient induction of human spinal LMNs, we combined a small molecule-based approach with the transduction of transcription factors (Figure 1A). Briefly, iPSCs were treated with 3 μM SB431542 (SB), a transforming growth factor β (TGF-β) signaling inhibitor; 3 μM CHIR9901 (CHIR), a GSK-3β (glycogen synthase kinase 3) inhibitor; and 3 μM dorsomorphin (DM), a BMP signaling inhibitor, for 7 days to induce an embryoid body (EB)-like state (Fujimori et al., 2017). Subsequently, Sendai viruses (SeV) carrying mouse transcription factors Lhx3, Ngn2, and Isl1 (SeV-Lhx3-Ngn2-Isl1), which play critical roles in LMN development, were transfected at a multiplicity of infection (MOI) of 5 (Goto et al., 2017).

Induction efficiency was determined based on the expression of HB9 and ISLET1 (Stifani et al., 2014). Cells were fixed and stained on day 7 of LMN differentiation; 71.1% ± 8.1% of cells were positive for HB9 and, 82.2% ± 7.8% were positive for ISLET1 (Figures 1B and 1C). High induction efficiency of around 80% was also confirmed by fluorescence-activated cell sorting (FACS) analysis of Hb9 promoter::mGreenLantern lentivirus-infected MNs (Figure S1). RNA sequencing (RNA-seq) analysis showed that the neurons had the characteristic expression profile of LMNs, in that high expression of pan-neuronal and LMN markers accompanied low expression of cortical neuron, microglia, oligodendrocyte, astrocyte, neural stem cell, and pluripotency markers (Figure 1D). Notably, we observed higher expression of CHAT (Choline Acetylase), which is required for the cholinergic activity of LMNs. (Wetts and Vaughn, 1994; Stifani et al., 2014). These trends were maintained until day 45 of differentiation. However, there was a decline in neuronal marker expression and an overall increase in glial marker expression at day 45 compared with day 14. We examined the induction efficiency at day 14 and 45 and discovered that the percentage of ISL1-positive cells declined from day 14 to day 45 (Figure S2). This decline of ISL1-positive cells indicates an increased proportion of unwanted cell types and thus explains the decreased neuronal marker expression in bulk mRNA-seq at day 45 (pan-neuronal and motor rows in

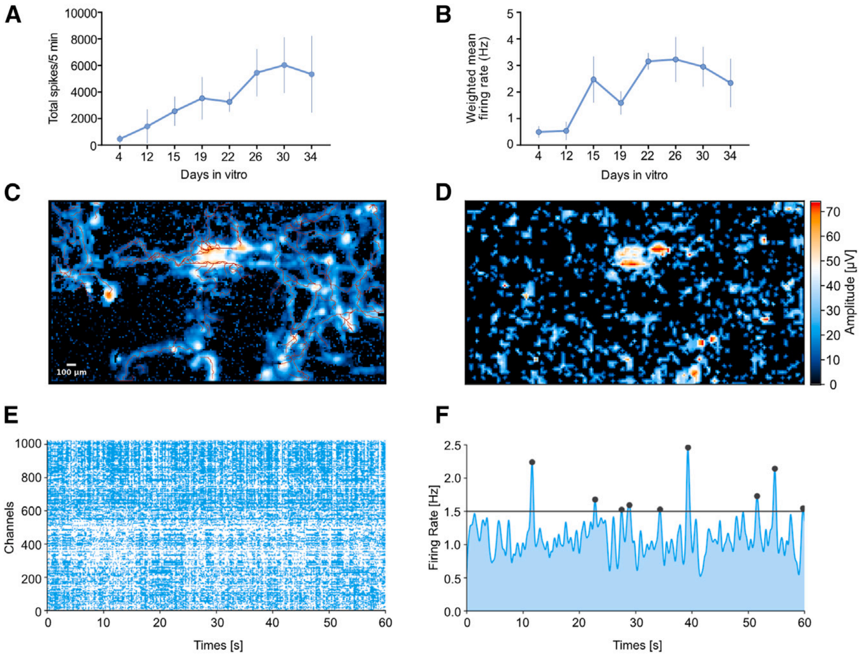

Figure 2. LMN showed spontaneous functional activity

(A) Total number of spikes that correspond to local field potential activity was measured within 5 min of MEA recording. Plot shows mean ± SEM from 3 independent wells.

(B) The weighted mean firing rates were computed as the average firing rates corrected for the number of active electrodes. Plot shows mean ± SEM from 3 independent wells.

(C) Axon tracking analysis by using the MaxOne MEA system, correspond to Table S2.

(D) Network activity map measured by MaxOne MEA, showing strong action potential activity.

(E) Representative raster plot from one of the active neuron sites; the plot above each raster plot shows each spike detected for each electrode over a 1 min recording period.

(F) Mean firing rate activity was measured over a 1 min recording period.

Figure 1D). We also observed higher expression of AIF1 (a microglial marker) in some cell lines, consistent with a study by Lituma et al., which suggested that AIF1 expression is important for the development of both microglia and excitatory neurons (Lituma et al., 2021). These expression profiles were confirmed by qPCR analysis from day 5 to day 30 (Figure S3). Regarding mRNA expression, endogenous expression of LHX3, NGN2, and ISL1 was minimal compared to the exogenous expression induced by SeV, which continued at least until day 45 (Figure S4). It is highly possible that endogenous gene expression here is suppressed by the abundant exogenous expression.

We also successfully derived LMNs using SeV carrying human transcription factors LHX3, NGN2, and ISL1 (SeV-LHX3-NGN2-ISL1 and SeV-LHX3-NGN2-ISL1-mEmerald) (Figure S5). Induction efficiency was comparable to that of mouse SeV-Lhx3-Ngn2-Isl1, reaching around 80% at day 14 (Figures S5A and S5B). Nonetheless we used mouse TFs (transcription factors) throughout the paper as the use of mouse TFs was well characterized in the previous report by Goto et al. (2017).

Next, we examined the regional character of the induced MNs. Regional association of MNs in the spinal cord is determined by the expression of *HOX* genes (Sagner and Briscoe, 2019). All *HOX* genes that determine regions in the spinal cord (*HOX*4-10) were upregulated compared to iPSCs (Figure 1E). The regional composition of induced LMNs, based on Hox gene expression patterns, was broad, ranging from the cervical to the thoracic lumbar spinal cord, with *HOX*4-10 genes being dominant compared to the lumbar and sacral spinal cord genes (*HOX*10-13) (Figure 1F).

To further characterize the induced LMNs, their spontaneous functional activity was tested. We plated them onto two independent MEA systems: Maestro and MaxOne. In the Maestro system, each well contains 16 electrodes, while, in the MaxOne system, each plate contains 26,400 electrodes, providing better coverage for measuring functional activity. After SeV induction, LMNs were cultured for 2 weeks before being plated into MEA. Functional measurements were taken over a period of 1 month for the Maestro system and at a single time point for the MaxOne system. We tracked the development of spontaneous activity and observed a progressive increase in the number of spikes and the weighted mean firing rate in the Maestro MEA (multi electrode array) system (Figures 2A and 2B). Using the axon tracking function, we observed the formation of long axons across the neuronal network, where the mean length of the longest axon was approximately 864 μm and the furthest distance from the initiation site was 920 μm (Figure 2C and Table S1). Additionally, the network activity map showed an active LMN culture, as demonstrated by the raster plot and mean firing rate activity (Figures 2D–2F, and Table S2). We conclude that these results indicate the LMNs are in a mature functional state, as demonstrated by robust neuronal firing activity—evidenced by mean firing rates and network burst activity—and supported by axon tracking data

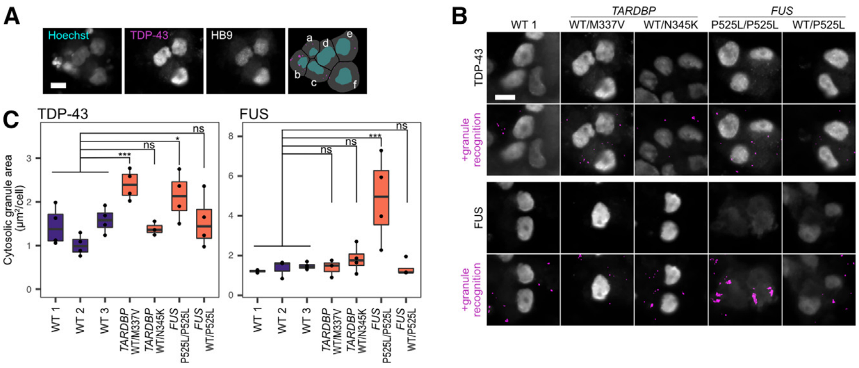

Figure 3. Spinal lower motor neurons derived from ALS-iPSCs exhibit distinct protein aggregation

(A) Representative images of the granule recognition strategy. Each cell was recognized based on Hoechst staining. The soma was defined by the collar function, and granules were recognized by the robust puncta function of IN Carta. The last image shows segmented cells with nuclei (cyan), soma (gray), and granules (pink). Mis-recognized dead cells or HB9-negative cells, such as a–c in the image, were removed from analysis by defining them using Phenoglyphs of IN Carta. In the figure a–c are dead cells with fragmented small nuclei and d–f are live positive cells. Scale bar, 10 μm.

(B) Representative images of TDP-43 and FUS granule recognition in each cell line. Scale bar, 10 μm.

(C) Mean cytosolic granule area per cell. Results were from three to four independent experiments. Two-tailed Dunnett's test was performed. *$p < 0.05$, **$p < 0.01$; ***$p < 0.001$; ns, non-significant.

showing a conduction velocity exceeding 0.5 m/s (Radivojevic and Rostedt Punga, 2023; Schenke et al., 2021; Xu et al., 2023).

LMNs derived from ALS-iPSCs show pronounced protein aggregation

To determine whether LMNs with ALS mutations exhibit pathological phenotypes, we used four ALS-iPSC lines carrying genetic mutations in either TDP-43 (*TARDBP*, TAR DNA binding protein 43) or FUS (fused in sarcoma). Aberrant cytoplasmic accumulation and aggregation of TDP-43 in MNs is a prevalent pathology in ALS patients, both in sporadic and familial cases with TDP-43 mutations (Sagner and Briscoe, 2019; Arai et al., 2006; Sreedharan et al., 2008; Lagier-Tourenne and Cleveland, 2009; Tamaoka et al., 2010). *FUS* mutations are another well-studied cause of familial ALS, where mutant FUS protein accumulates and aggregates aberrantly in the cytoplasm (Vance et al., 2009; Kwiatkowski et al., 2009).

We performed immunocytochemistry (ICC) on LMNs derived from the following cell lines to determine whether ALS LMNs retain these pathological phenotypes such as TDP-43 and FUS aggregation in the cytoplasm. The ALS-iPSC lines used were *TARDBP*$^{\mathrm{WT/M337V}}$ (A3411, derived from an ALS patient), *TARDBP*$^{\mathrm{WT/N345K}}$ (SM4-4-5, derived from an ALS patient), *FUS*$^{\mathrm{P525L/P525L}}$ (FUS-008-1-E6, isogenic mutated cell line of WT 1; wild type), and *FUS*$^{\mathrm{WT/P525L}}$ (FUS-008-1-G2, isogenic mutated cell line of WT 1) (Table S3). These lines were induced to differentiate into LMNs using the protocol described earlier. On day 7 of differentiation, cells were fixed and immunostained and images were analyzed using machine learning for single-cell level ICC image analysis to exclude dead and HB9 or ISL1-negative cells (Figure 3A).

As expected, *TARDBP*$^{\mathrm{WT/M337V}}$ LMNs had more aggregated TDP-43 in their cytoplasm than control WT cell lines (Figures 3B and 3C). For the ALS patient with the *TARDBP*$^{\mathrm{N345K}}$ mutation, it has been reported that, in the autopsy, TDP-43 aggregation or mis-localization was not observed in LMNs, but rather in astrocytes (Takeda et al., 2019). Our findings for *TARDBP*$^{\mathrm{WT/N345K}}$ were consistent with the patient's symptoms, showing a lack of cytosolic granules in LMNs. On the other hand, although ALS patients with *FUS* mutations typically do not show cytosolic TDP-43 aggregation in MNs, *FUS*$^{\mathrm{P525L/P525L}}$ LMNs showed a significant increase in TDP-43 aggregation in the cytosol (Lagier-Tourenne and Cleveland, 2009).

We also analyzed TDP-43 mis-localization using the cytoplasm-to-nucleus mean intensity ratio for each cell (Figure S6). While ALS patients with *TARDBP* mutations (excluding *TARDBP*$^{\mathrm{N345K}}$) often exhibit TDP-43 depletion from the nucleus and accumulation in the cytosol, our analysis showed little difference between WT and ALS LMNs in terms of fluorescence intensity (Figure S6 top right panel).

We also examined FUS aggregation and accumulation in the cytoplasm. As expected, *FUS*$^{\mathrm{/P525L/P525L}}$ LMNs showed significantly more FUS aggregation and accumulation in the cytoplasm (Figures 3C and S6 bottom right panel). Although *FUS*$^{\mathrm{WT/P525L}}$ LMNs did not show a significant increase of FUS granules in the cytoplasm, we detected mis-localization based on the cytoplasm-to-nucleus mean intensity ratio. The lack of some disease phenotypes of *FUS*$^{\mathrm{WT/P525L}}$ may be explained by the fact that it is a heterozygous *FUS* mutation.

The observed aggregation and mis-localization of TDP-43 and FUS in the ALS cell lines indicate that our LMN induction method and single-cell-based image analysis are suitable for disease research.

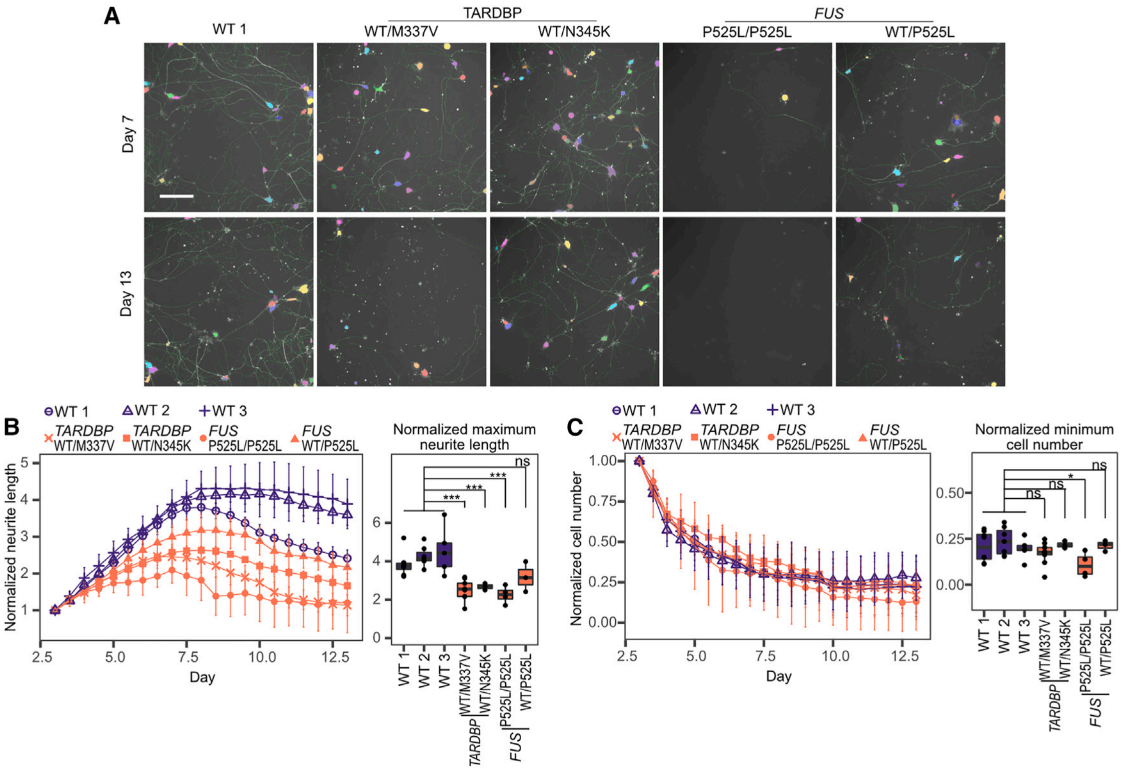

Figure 4. Spinal lower motor neurons derived from ALS-iPSCs show reduced outgrowth of neurites

(A) Representative image of neurite (green) and soma (randomly assigned color) recognition based on EGFP florescence on days 7 and 13. Scale bar, 160 μm.

(B) Total neurite length of each well was normalized by dividing by the corresponding neurite length at day 3. n = 3–5 independent experiments. Normalized maximum neurite length was compared and tested using Dunnett's test.

(C) Total cell number of each well was normalized by dividing by the cell number on day 3. n = 3–5 independent experiments. Normalized minimum cell number was compared and tested using Dunnett's test. For (B) and (C), error bars represent standard error. $^{*}p < 0.05$; $^{**}p < 0.01$; $^{***}p < 0.001$; ns, non-significant.

Bulk analysis shows reduced neurite outgrowth in LMNs derived from ALS-iPSCs compared to WT controls

Next, we investigated the vulnerability of LMNs derived from ALS-iPSCs, as MN degeneration is a key symptom in ALS patients. To assess the phenotype of induced MNs from ALS-iPSCs, we examined neurite growth (Fujimori et al., 2018; Morimoto et al., 2023). Cell images were acquired every 12 h from day 3 to day 13 to evaluate neurite length and cell number. We used a Sendai virus that transduced EGFP along with transcription factors Lhx3, Ngn2, and Isl1 to allow for clearer detection of neurites (Figures 1A and 4A).

Overall, our LMNs retained the expected phenotypes based on their genotype. We compared the normalized maximum neurite length and the normalized minimum cell numbers (Figure 4B right and 4C right). The maximum neurite length was significantly shorter for ALS cell lines, except for the *FUS*$^{\mathrm{WT/P525L}}$ cell line. *FUS*$^{\mathrm{WT/P525L}}$ cells, with a heterozygous *FUS* mutation, had a maximum neurite length comparable to WT cell lines. We also replicated the reduced outgrowth of ALS LMN neurites using human transcription factors SeV and SeV-LHX3-NGN2-ISL1-mEmerald with WT 1 and *TARDBP*$^{\mathrm{WT/M337V}}$ (Figures S5C and S5D).

Although *TARDBP*$^{\mathrm{WT/M337V}}$, *TARDBP*$^{\mathrm{WT/N345K}}$, and *FUS*$^{\mathrm{P525L/P525L}}$ had shorter neurites, there were no significant differences in the normalized minimum cell number, except for *FUS*$^{\mathrm{P525L/P525L}}$ (Figure 4C). However, ALS cell lines *TARDBP*$^{\mathrm{WT/M337V}}$ and *FUS*$^{\mathrm{P525L/P525L}}$ died faster than WT cell lines according to microscopic observation. Additionally, when we evaluated apoptosis with immunostaining of cleaved caspase-3 (CC3), we found that *TARDBP*$^{\mathrm{WT/M337V}}$ and *FUS*$^{\mathrm{P525L/P525L}}$ had a greater granule area and more CC3-positive cells compared to WT lines, indicating stronger apoptotic signals (Figure S7).

We observed an increase in cellular debris over time, which was hard to distinguish from live cell bodies by the

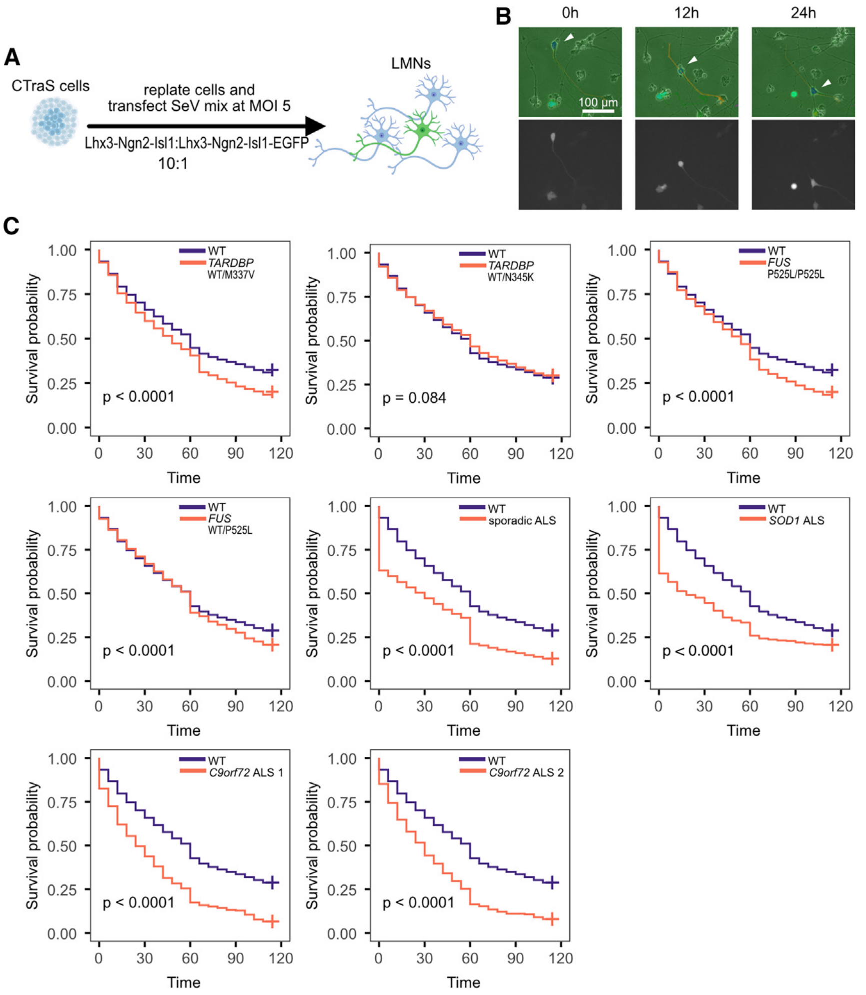

Figure 5. Single-cell tracking and survival analysis revealed that ALS-iPSC-induced LMN dies faster than WT controls

(A) Experimental scheme.

(B) Representative image of cell tracking. The white arrowhead indicates the tracked cell. (Top) EGFP images are overlaid on phase contrast images. (Down) EGFP images.

(C) Survival curves were compared between ALS cell lines and WT control cell lines (201B7 and WD39). *p* values were calculated by the log rank test corrected with the Benjamini-Hochberg procedure.

analysis software. This debris was misrecognized as cells by automated image analysis, making accurate cell counting difficult. We hypothesized that analyzing only cells that could be tracked from the start to the end of the experiment or until cell death would provide a clearer survival analysis. Therefore, to clearly evaluate cell survival probability, we developed a method for single-cell resolution analysis.

Cell vulnerability more accurately evaluated by single-cell tracking and survival analysis

For accurate single-cell tracking, it is essential to detect target cells precisely. To avoid overcrowding of EGFP-positive cells, we mixed SeV-L-N-I and SeV-L-N-I-EGFP cells at a 10:1 ratio (Figures 5A and 5B). The starting point (0 h) of survival analysis was set at 4 days post-Sendai virus transfection. To minimize the possibility of cells moving out of the field of view, we acquired 5 × 5 tiling images.

As expected, *TARDBP*$^{WT/M337V}$ and *FUS*$^{P525L/P525L}$ LMNs were significantly more likely to die compared to healthy LMNs, whereas the survival curve of *TARDBP*$^{WT/N345K}$ was not different from that of healthy cell lines (Figure 5C). For *FUS*$^{WT/P525L}$, although we did not observe significant differences in bulk analysis earlier, we observed a significant difference in the survival curve. We further tested 4 additional ALS cell lines to confirm our results. Using this method, we successfully confirmed the vulnerability of cells carrying other ALS mutations and cells derived from sporadic ALS patients (Figure 5C).

DISCUSSION

We have demonstrated the high efficiency of a novel spinal LMN induction protocol. The single-cell-based evaluation method showed that induced MNs with ALS mutations presented phenotypes consistent with major characteristics of the disease as established in previous reports, such as cytoplasmic TDP-43/FUS aggregation and neurite vulnerability. Earlier studies using Sendai virus to derive MNs reported an induction efficiency of about 30%

Table 1. Comparison of MN induction methods

Reference	Efficiency (%)	Total days	Differentiation approach	Key molecules	Disease recapitulation
Du et al., 2015	95 (/DAPI))	28	small molecule	N2, B27, CHIR, DMH1, SB, RA, PMN, VPA, BDNF, CNTF, IGF-1, compound E	SOD, neurite fragmentation
Goto et al. (2017)	30 (/DAPI)	21	Sendai virus	SB, dorsomorphin, SeV-TFs, RA, SAG, N2, B27, BDNF, GDNF, NT-3	SOD, TDP-43 aggregation
Sepehrimanesh and Ding (2020)	>90 (/TUBB+)	39	lentivirus	Lentivirus-TFs, β-ME, N2, NEAA, heparin, bFGF, EGF, B27, forskolin, BDNF, GDNF, NT-3	not mentioned
Present paper	80 (/DAPI)	21	Sendai virus	CHIR, SB, dorsomorphin, SeV-TFs, B27, BDNF, GDNF, RA, AA, DAPT, PD	FUS, TDP-43 aggregation, neurite length, survival

(Goto et al., 2017). By contrast, our method improved efficiency to 70%–80% by transforming iPSCs into a chemically transitional EB-like state (CTraS) with three small molecules—SB, DM, and CHIR99021—before SeV treatment. CTraS accelerates differentiation by inhibiting TGF-β/BMP and activating Wnt (wingless type MMTV integration site family) signaling (Fujimori et al., 2017). This paper demonstrated that these molecules work well as a pretreatment for transcription factors and virus-based transcription factor induction. Although other protocols achieve higher purity of MNs, the speed and efficiency with which it produces LMNs (70%–80%) is a significant advantage of our protocol (Table 1) (Du et al., 2015; Sances et al., 2016; Sepehrimanesh and Ding, 2020).

There were interesting discrepancies in the neurite analysis and survival analysis of *TARDBP*$^{WT/N345K}$ and *FUS*$^{WT/P525L}$ (Figures 4B and 5C). *TARDBP*$^{WT/N345K}$ did not have a lower survival probability despite showing shorter neurites, while *FUS*$^{WT/P525L}$ did not show shorter neurites despite having a lower survival probability. These discrepancies may be due to a limitation in the protocol or could reflect differences in the molecular pathways by which these mutations affect LMNs. For example, the WT/N345K mutation of *TARDBP* may work "backwards" in that it first affects neurites, and the WT/P525L mutation of *FUS* may affect "forwards" in that it is lethal for the cell but has a delayed effect on the neurites. Investigating these differences may reveal more detailed molecular mechanisms underlying ALS pathology.

Our rapid differentiation and scoring methods have potential to accelerate drug screening (Okano and Morimoto, 2022; Bonaventura et al., 2021) and facilitate reverse translational research by comparing the therapeutic effects of candidate drugs in both *in vitro* and *in vivo* models (Morimoto et al., 2023). Over the past decade, several drugs have entered clinical trials based on preclinical tests or screenings using MNs derived from iPSCs or embryonic stem cells (ESCs) (Table 2). However, these screenings predominantly rely on a limited number of iPSC lines with genetic mutations, despite the fact that sporadic ALS cases account for approximately 90% of the total. This is partly because screenings using genetically mutated cell lines can produce clearer results with fewer lines. In contrast, drug screening for sporadic ALS requires a larger and more diverse set of cell lines due to the heterogeneous nature of the disease, which makes achieving clear results more challenging with only a few lines. Our rapid differentiation and scoring methods could broaden the scope of drug screening by including a wider range of sporadic ALS cell lines.

Furthermore, our methods have the potential to enable personalized drug screening. ALS symptoms progress rapidly, rendering daily physical activities impossible within 1–2 years. Therefore, the rapid establishment of patient-derived iPSCs and drug screening of MNs is essential for precision medicine. Although there are many challenges to the practical application of personalized medicine using iPSCs (Shin et al., 2018; Novelli et al., 2022; Palasantzas et al., 2023; Nguyen et al., 2022), our method can shorten the time required for drug screening and contribute to practical applications.

Single-cell-based analysis is a powerful tool in the study of heterogeneous cell populations (Mattiazzi Usaj et al., 2021). In our study, we expanded the application of single-cell tracking (Shin et al., 2018) to cell survival analysis to accurately evaluate the cellular vulnerability of ALS patients using long-term live imaging and single-cell tracking of iPSC-derived spinal LMNs. Further associating the morphological data of each cell with multi-omics expression data would be interesting (Watson et al., 2021; Park et al., 2022). For ALS studies using iPSC-derived LMNs, associating phenotypic data, such as cell morphology and ICC images, with omics data could help analyze differences between the most affected and less affected cells in an isogenic cell line and identify epigenetic differences among cells with identical genomic backgrounds.

In this study, we used a limited number of ALS-iPSC lines with genetic mutations. Although our induced MNs successfully reproduced typical ALS phenotypes and partial

Table 2. Drug developments based on induced MNs

Reference	Efficiency (%) Isl1 or HB9	Total days	Differentiation approach	Rescued phenotype	Number of tested drugs	Number of tested ALS patient-derived cell line	Features of tested cell lines	Drug
Donnelly et al. (2013)	30–40	40–45	small molecule	C9orf72 RNA level, nuclear GGGGCC RNA foci, Glutamate exposure susceptibility	5 (antisense oligo)	4	C9orf72	antisense oligo
Wainger et al. (2014)	N.A. (FACS purified)	52	small molecule	hyperexcitability	1	2	SOD1	ezogabine (retigabine)
Naujock et al. (2016)	60-67 (/DAPI)	>42	small molecule	spontaneous activity, ion channel imbalance	1	6	SOD1, FUS	4-aminopyridine (4AP)
Imamura et al. (2017)	60 (/DAPI)	14	TFs polycistronic vector	misfolded SOD1, survival	1,416	1	SOD1	bosutinib
Guo et al. (2017)	80 (/DAPI)	32–38	small molecule	FUS mis-localization, hyperexcitability, axonal transport	1	3	FUS	ACY-738/ Tubastatin A
Fujimori et al. (2018)	65 (/DAPI)	60	small molecule	FUS and TDP-43 aggregation/mis-localization, neurite length, LDH leakage, CC3	1,232	4	TDP-43, FUS	ropinirole
Thams et al. (2019)	40 (/DAPI)	31	small molecule	survival (under ER stress)	1,275	1	SOD1 (edited ESC)	TUDCA

correlation between clinical drug response and *in vitro* assays was observed in another study using this induction method, it is not clear whether the severity of the phenotype correlates with the patient's clinical progression (Morimoto et al., 2023). It should also be noted that the exogenous expression of transcription factors Lhx3, Ngn2, and Isl1 may have some effect on the induced MNs' disease phenotype. Future extensive studies analyzing relationships between patients' pathological phenotypes, including sporadic cases, and the corresponding induced MN phenotypes are necessary. Recent advances in large libraries of iPSCs from ALS patients with clinical records, such as Answer ALS (Baxi et al., 2022) and JaCALs (Nakamura et al., 2023), will help evaluate the usefulness and precision of ALS disease modeling approaches.

EXPERIMENTAL PROCEDURES

Research ethics

This study was conducted in accordance with the Declaration of Helsinki and was approved by the ethics committee of Keio University School of Medicine (approval no. 20080016).

Human feeder-free iPSC culture

Feeder-free iPSCs were maintained in StemFit AK02N medium (Ajinomoto, Tokyo, Japan). Cells were dissociated using 0.5 × TripLE Select (Thermo Fisher Scientific, Waltham, MA) and seeded at 0.3–1 × 10^4 cells/well in six-well plates treated with 2 μL/mL iMatrix-511 (Matrixome, Osaka, Japan) (Laminin 511E8; FUJIFILM Wako Pure Chemical Corp., Tokyo, Japan). Y27632 (10 μM; Nacalai, Kyoto, Japan) was added only for the first day. The medium was changed every other day. The cell lines used in this study are described in Table S3 (Takahashi et al., 2007; Okita et al., 2011; Imaizumi et al., 2012; Egawa et al., 2012; Leventoux et al., 2020).

LMN induction from iPSCs using Sendai viruses

LMNs were induced from iPSCs via a chemically transitional EB-like state (CTraS) (Fujimori et al., 2017) and infection with SeV-L-N-I (ID Pharma, Tsukuba, Japan), SeV-L-N-I-EGFP (ID Pharma) (Goto et al., 2017), or SeV-LHX3-NGN2-ISL1 (Repli-tech Co., Ltd, Tokyo, Japan). Briefly, iPSCs were seeded in StemFit AK02N (Ajinomoto) with 10 μM Y27632 (Nacalai) and 2 μL/mL iMatrix-511 (Matrixome) and cultured for 5 days at 37°C under 5% CO_2. The medium was changed the following day to remove Y27632 and then every 2 days thereafter.

To induce an EB-like state, the medium was changed to a chemical induction medium (StemFit AK02N with 3 μM SB [Sigma-Aldrich, St. Louis, MO], 3 μM CHIR [Cayman, Ann Arbor, MI], and 3 μM DM [Santa Cruz, Dallas, TX]) for 7 days (Fujimori et al., 2017). The medium was changed daily during this period.

After 7 days, iPSC colonies were dissociated into single cells using 0.5 × TripLE Select and seeded onto plates coated with 0.0001% poly-L-lysine (PLL) (Sigma-Aldrich), 10 μL/mL Matrigel (Thermo Fisher Scientific), and 6.4 μL/mL iMatrix-511 (Matrixome). PLL was diluted in distilled water, and wells were kept overnight at 37°C. After removing PLL, wells were treated with the Matrigel and iMatrix-511 mixture in phosphate-buffered saline (PBS) and incubated at 37°C for at least 1 h. The mixture was removed just before plating the cells. The same concentration of coating reagents was used for both 96-well and 12-well formats.

The MN medium consisted of KBM neural stem cell medium (KOHJIN BIO, Saitama, Japan) supplemented with 2% B27 ((Thermo Fisher Scientific), penicillin (100 U/mL)-streptomycin (100 μg/mL) (Thermo Fisher Scientific), 200 μM ascorbic acid, 10 ng/mL brain-derived neurotrophic factor (R&D Systems, Minneapolis, MN), 10 ng/mL glial cell-derived neurotrophic factor (Alomone Labs, Jerusalem, Israel), 10 μM DAPT (Sigma-Aldrich), and 10 μM Y27632. SeV was applied at an MOI of 5. Y27632 was removed from the MN medium after 1 day. The medium was changed on days 1, 3, 4, 7, 10, and 13 and every three days thereafter. DAPT was added until day 7. On days 4 and 7, 2 μM PD0332991 (Sigma-Aldrich) was added to remove proliferating cells. If there was no change in the medium components, only half the volume of MN medium was exchanged to avoid damaging LMNs. Cells were incubated at 37°C with 4% O_2 and 5% CO_2 until day 7 for ICC or day 13 for neurite analysis and single-cell tracking (Santilli et al., 2010).

For ICC, cells were seeded in 96-well plates at 1 × 10^5 cells/well and infected with SeV-L-N-I. For neurite length analysis, cells were seeded in 12-well plates at 5 × 10^5 cells/well and infected with SeV-L-N-I-EGFP. For single-cell tracking, SeV-L-N-I and SeV-L-N-I-EGFP were mixed at a 10:1 ratio and cells were infected at an MOI of 5. Cells were seeded at 6 × 10^5 cells/well in 12-well plates.

RNA-seq and analysis

Total RNA was isolated from LMNs on days 14 and 45 using an RNeasy mini kit (QIAGEN, Hilden, Germany) with DNase I treatment. RNA libraries for RNA-seq were prepared using a Nextera XT DNA library prep kit (Illumina, San Diego, CA) following the manufacturer's protocols. Raw fastq files were trimmed to remove low-quality bases and adapters using fastp v.0.23.2 (Chen et al., 2018) and processed for further analyses. To generate TPM, salmon v.1.7.0 was used (Patro et al., 2017). The transcript index was created from the reference GRCh38 genome annotation (GENCODE release 39) to quantify gene expression levels. DESeq2 was used to identify differentially expressed genes (Love et al., 2014) with a cutoff of 0.05 for Benjamini-Hochberg-adjusted *p* values and a cutoff of 0.25 for the log2 fold change ratio.

For the analysis of exogenous expression in Figure S4, reads were mapped to GRCh38 release 84 version of *H. sapiens* using HISAT2 (version 2.1.0) with relaxed mismatch penalty (scoring option; –mp 1,0) (Kim et al., 2019). Resulting sam files were converted into bam files using samtools version 1.9 (Danecek et al., 2021). The data were investigated using IGV (Ingetrative Genomic Viewer) (Robinson et al., 2011).

Quantitative reverse-transcription PCR

Total RNA was isolated using an RNeasy Mini Kit (QIAGEN) and treated with DNase I. cDNA was prepared using an iScript cDNA synthesis kit (Bio-Rad, Hercules, CA). Quantitative reverse-transcription PCR was performed using Premix Ex Taq II (Takara Bio Inc., Shiga, Japan) on a ViiA 7 real-time PCR system (Thermo Fisher

Scientific). qPCR primers are listed in Table S4. Relative expression was calculated using the ΔΔCT method. ACTB (actin beta) was used as the internal control to calculate ΔCT, and the control condition to calculate ΔΔCT was day 0 WT 3 (414C2).

High-density microelectrode array recording by MaxOne

MaxWell Biosystems high-density microelectrode arrays (HD-MEAs) (MaxWell Biosystems, Zürich, Swiss) were used to assess the electrophysiological activity of MNs. Prior to HD-MEA experiments, MNs were differentiated for two weeks post-SeV transduction in MN culture medium in regular tissue-culture plates (see method on LMN induction from iPSCs using Sendai viruses). On the day of plating, MNs were dissociated with Accutase and counted using the Countess system (Thermo Fisher Scientific).

MaxOne HD-MEA chips were incubated with 1% Tergazyme solution for 2 h at room temperature and washed with water, followed by submersion in 70% ethanol for sterilization for approximately 1 h. HD-MEA chips were washed with sterile water three times, and then 1 mL of complete culture media consisting of BrainPhys medium with SM1 (STEMCELL Technologies, Vancouver, Canada) was added to prime the chips. HD-MEA chips were incubated for 2 days in a humidified cell culture incubator at 37°C with 5% CO_2.

On the day of cell plating, the culture media were removed and chips were washed three times with sterile water prior to coating with 0.1% polyethyleneimine (PEI, Sigma-Aldrich). Chips were placed back in the incubator for 1 h. PEI was then removed, and the chips were washed three times with sterile water and allowed to dry in the biosafety cabinet for 1 h. Laminin was then applied to the chips, which were placed back in the incubator until the cells were ready to be seeded. Laminin coating was removed, and 50 μL of MN suspension was pipetted into the middle section of the chip at a density of 50,000 cells per chip. After 1 h of incubation, 600 μL of MN culture medium was added to each chip. The next day, 50% of the medium in each chip was replaced with fresh culture medium. Media changes were performed every 3 days by removing 50% of the medium and replenishing it with an equal volume of fresh culture medium. HD-MEA recordings were performed on day 14 post-plating. The MaxWell Biosystems recording unit was sterilized with 70% ethanol, placed in the biosafety cabinet, and allowed to dry for 30 min. The recording unit was then transferred to an incubator for at least 2 h for temperature equilibration prior to recording.

To assess neuronal activity across the entire electrode array, the "Activity Scan Assay" module in the MaxLab Live software was used. The network assay module was then used to assess network activity or axonal features. Network electrical activity was recorded by selecting a subset of electrodes with the highest firing rate from the corresponding chip's activity scan and measured simultaneously for 5 min.

Microelectrode array recording by Maestro

Forty-eight well CytoView MEA plates (Axion Biosystems, Atlanta, GA) were coated with 0.1% PEI at room temperature for 1 h, washed with sterile water, and dried overnight in a sterile environment. Two weeks after SeV transduction, MNs were dissociated with Accutase, resuspended at a count of 50,000 cells in 5 μL of MN media with 10 μg/mL Laminin, and drop-pipetted to the center of the electrode area. After 1 h, modified MN media (substituting KBM neural media with BrainPhys media, STEMCELL Technologies) were added drop-wise to the sides of the well. Spontaneous network activity was then recorded using the Maestro MEA system (Axion Biosystems). Neural signals were sampled at 12.5 kHz and high-pass filtered (200 Hz–3 kHz), and a threshold based on six standard deviations above noise level was set by Axion Integrated Studio Software (AxIS). The burst detector was configured to detect network bursts with a maximum interspike interval of 100 ms, a minimum of 50 spikes, and a minimum of 35% participating electrodes. After recording, the data were analyzed using the Neurostatistics compiler (Axion Biosystems).

ICC

Cells were fixed in 4% paraformaldehyde in PBS for 35 min at room temperature. Following fixation, cells were blocked with 10% goat serum and 0.3% Triton X-100 for 1 h at room temperature. Cells were then incubated overnight at 4°C with primary antibodies described in Table S5. Antibodies were diluted with 5% fetal bovine serum (FBS) in PBS. The cells were rinsed with PBS and incubated with species-specific Alexa Fluor 488-, Alexa Fluor 555-, or Alexa Fluor 647-conjugated secondary antibodies (1:2,000; Invitrogen, Thermo Fisher Scientific) mixed with bisbenzimide H (Hoechst) 33258 (Sigma-Aldrich) to counterstain nuclei. Images were obtained using the IN Cell Analyzer 6000 with a ×60 objective lens (Cytiva, Tokyo, Japan). Cell numbers analyzed for each experiment are listed in Table S6.

Image analysis of ICC

Images acquired by the IN Cell Analyzer 6000 were analyzed using IN Carta (Cytiva) with SINAP and Phenoglyphs modules. Each cell was analyzed based on nuclear staining. The somatic area was defined using the collar function, and granular objects of TDP-43, FUS, and CC3 staining were detected using the robust puncta function. Phenoglyphs was used to cluster recognized cells into various groups. The machine was trained to determine whether each group of cells was alive or dead based on nuclear staining and categorized as positive or negative based on marker gene (HB9/ISLET1) staining. Dead cells were identified based on the morphology of nuclear staining, where the nuclei of dead cells undergoing apoptosis exhibit a compact shape and higher intensity due to fragmented chromatins. After applying the classification to all images, the differentiation efficiency of live cells was calculated. Cells that were alive and positive for marker genes were used for further analysis, including the analysis of mis-localization of TDP-43 and FUS. CC3-positive cells were defined as cells with CC3 granules greater than 20 μm^2.

Flow cytometry

The cultured MNs were transfected with lentivirus vector backbone designed to express mGreenLantern (Campbell et al., 2020) from an Hb9 promoter. The cells were dissociated using dissociation buffer including papain and Accutase for 20 min, and clumps or debris were removed with a 40 μm cell strainer.

The number of mGreenLantern-positive cells in 200 μL PBS with 2% FBS was measured using a CytoFLEX S flow cytometer

(Beckman Coulter, Indianapolis, IN, USA) with CytExpert software, and 50,000 cell events were recorded per sample. Debris and doublets were filtered out. Gates were set based on appropriate unstained samples.

Automated time-lapse live imaging

Images were acquired using BioStation CT (Nikon) from day 3 to day 13. For neurite length analysis, images were automatically acquired every 12 h with six image points per well using a ×10 objective lens for phase contrast and GFP images. For single-cell tracking, 5 × 5 tiling images were acquired every 6 h for each well.

Cell tracking and image analysis

Image analysis was performed using the CL-Quant software (Nikon). Nikon developed a specialized analysis recipe for our dataset. The recipe is available upon request. To analyze the morphological characteristics of cells in culture, morphological filters, including background subtraction, thresholding, and line segmentation, were designed to identify and quantify various features in the raw image, such as cell body number, neurite length, cell body size, compactness, and intensity. For node detection, a binarization combination of the cell body filter and neurite filter was used to detect overlapping regions between the neurite and cell body, which were then used to identify nodes in raw images. After optimizing the filters to detect the individual cells, tracking was performed using the filter for cell bodies of ≥100 pixels.

The settings used in CL-Quant for this tracking were as follows: minimum object size, −1; maximum object size, 999999; maximum search range, 300; split threshold, 0.90; merge threshold, 0.70; minimum trajectory length, 1; object split, ignore split; object merge, merge with partition; enable lineage, off; enable robust measurement, off; object-to-object overlap, off; remove short trace when merging, off. Images acquired between day 4 and day 10 were used for single-cell tracking analysis.

Statistics

Statistical tests and sample sizes (n) are indicated in the figure legends. Statistical analyses were performed using GraphPad software (v.8.2.1) or R. Significance levels were set as follows: *$p < 0.05$, **$p < 0.01$, ***$p < 0.001$, and ns for non-significant.

RESOURCE AVAILABILITY

Lead contact

Requests for further information and resources should be directed to and will be fulfilled by the lead contact, Satoru Morimoto (satoru_morimoto@keio.jp).

Materials availability

Materials are available from the corresponding authors upon request.

Data and code availability

The data that support the findings of this study are available from the corresponding author upon reasonable request. RNA-seq data have been deposited at the Gene Expression Omnibus (GEO) database with accession number GSE229095 and are publicly available as of the date of publication.

ACKNOWLEDGMENTS

The authors would like to thank all the members of the H.O. laboratory for their encouragement and kind support. We would also like to thank Shinya Yamanaka, Keisuke Okita, and Makoto Nakagawa (Kyoto University) for kindly providing 201B7, 1210B2, and 414C2; Haruhisa Inoue (Kyoto University) for kindly providing A3411 and A21412; and Takeda Pharmaceutical Company for kindly providing FUS-008-1-E6 and FUS-008-1-G2 iPSC lines. S.M. reports grants supports from the Japan Society for the Promotion of Science (JSPS) (KAKENHI grant no. JP21H05278, JP22K15736, and 22K07500), the Japan Agency for Medical Research and Development (AMED) (grant no. JP23bm1123046 and JP23kk0305024), The Kanae Foundation for the Promotion of Medical Science, the Uehara Memorial Foundation, the Yukihiko Miyata Memorial Trust for ALS Research, Okasan-Kato Foundation Research Grant, Yoshio Koide Grant, Japan ALS Association, Daiichi Sankyo Foundation of Life Science, UBE Academic Foundation, and the Kato Memorial Trust for Nambyo Research during the conduct of the study. H.O. has grant supports from JSPS (KAKENHI grant no. JP20H00485, JP21F21410, JP21H05273, and JP22KF0 333) and AMED (grant no. JP22bm0804003, JP20ek0109395, JP20ek0109329, JP21wm0425009, and JP23bm1423002). The funding sources had no role in the analysis. Disclosure forms provided by the authors are available with the full text of this article at Stem Cell Reports. We thank Mitchell Arico from Edanz (https://jp.edanz.com/ac) for editing a draft of this manuscript.

AUTHOR CONTRIBUTIONS

S.M. designed the protocol of cell culture. F.O., S.N., and S.M. performed the experiments, prepared the samples for RNA-seq, performed the immunostaining, and imaged the cells. S.M. and S.S. analyzed the data and wrote the manuscript. S.S. performed the RNA-seq analysis. K.H.U. did the functional assay. A.N., N.S., M.A., and Y. Takeshita established iPSCs. S.M. and H.O. provided a grant for the study. Y. Tomari supervised the research and corrected the manuscript. H.O. corrected the manuscript and oversaw the research program. All authors have read and agreed to the published version of the manuscript.

DECLARATION OF INTERESTS

H.O. reports grants and personal fees from K Pharma Inc and SanBio Co. Ltd., outside the submitted work.

SUPPLEMENTAL INFORMATION

Supplemental information can be found online at https://doi.org/10.1016/j.stemcr.2024.11.007.

Received: April 7, 2023
Revised: November 13, 2024
Accepted: November 18, 2024
Published: December 19, 2024

REFERENCES

Arai, T., Hasegawa, M., Akiyama, H., Ikeda, K., Nonaka, T., Mori, H., Mann, D., Tsuchiya, K., Yoshida, M., Hashizume, Y., and Oda, T. (2006). TDP-43 is a component of ubiquitin-positive tau-negative inclusions in frontotemporal lobar degeneration and amyotrophic lateral sclerosis. Biochem. Biophys. Res. Commun. *351*, 602–611. https://doi.org/10.1016/j.bbrc.2006.10.093.

Baxi, E.G., Thompson, T., Li, J., Kaye, J.A., Lim, R.G., Wu, J., Ramamoorthy, D., Lima, L., Vaibhav, V., Matlock, A., et al. (2022). Answer ALS, a large-scale resource for sporadic and familial ALS combining clinical and multi-omics data from induced pluripotent cell lines. Nat. Neurosci. *25*, 226–237. https://doi.org/10.1038/s41593-021-01006-0.

Bonaventura, G., Iemmolo, R., Attaguile, G.A., La Cognata, V., Pistone, B.S., Raudino, G., D'Agata, V., Cantarella, G., Barcellona, M.L., and Cavallaro, S. (2021). iPSCs: A Preclinical Drug Research Tool for Neurological Disorders. Int. J. Mol. Sci. *22*, 4596. https://doi.org/10.3390/ijms22094596.

Campbell, B.C., Nabel, E.M., Murdock, M.H., Lao-Peregrin, C., Tsoulfas, P., Blackmore, M.G., Lee, F.S., Liston, C., Morishita, H., and Petsko, G.A. (2020). mGreenLantern: a bright monomeric fluorescent protein with rapid expression and cell filling properties for neuronal imaging. Proc. Natl. Acad. Sci. USA *117*, 30710–30721. https://doi.org/10.1073/pnas.2000942117.

Castillo Bautista, C.M., and Sterneckert, J. (2023). Progress and challenges in directing the differentiation of human iPSCs into spinal motor neurons. Front. Cell Dev. Biol. *10*, 1089970. https://doi.org/10.3389/fcell.2022.1089970.

Chen, S., Zhou, Y., Chen, Y., and Gu, J. (2018). fastp: an ultra-fast all-in-one FASTQ preprocessor. Bioinformatics *34*, i884–i890. https://doi.org/10.1093/bioinformatics/bty560.

Danecek, P., Bonfield, J.K., Liddle, J., Marshall, J., Ohan, V., Pollard, M.O., Whitwham, A., Keane, T., Mccarthy, S.A., Davies, R.M., and Li, H. (2021). Twelve years of SAMtools and BCFtools. GigaScience *10*, giab008. https://doi.org/10.1093/gigascience/giab008.

Donnelly, C.J., Zhang, P.W., Pham, J.T., Haeusler, A.R., Mistry, N.A., Vidensky, S., Daley, E.L., Poth, E.M., Hoover, B., Fines, D.M., et al. (2013). RNA Toxicity from the ALS/FTD C9ORF72 Expansion Is Mitigated by Antisense Intervention. Neuron *80*, 415–428. https://doi.org/10.1016/j.neuron.2013.10.015.

Du, Z.W., Chen, H., Liu, H., Lu, J., Qian, K., Huang, C.L., Zhong, X., Fan, F., and Zhang, S.C. (2015). Generation and expansion of highly pure motor neuron progenitors from human pluripotent stem cells. Nat. Commun. *6*, 6626. https://doi.org/10.1038/ncomms7626.

Egawa, N., Kitaoka, S., Tsukita, K., Naitoh, M., Takahashi, K., Yamamoto, T., Adachi, F., Kondo, T., Okita, K., Asaka, I., et al. (2012). Drug Screening for ALS Using Patient-Specific Induced Pluripotent Stem Cells. Sci. Transl. Med. *4*, 145ra104. https://doi.org/10.1126/scitranslmed.3004052.

Fujimori, K., Ishikawa, M., Otomo, A., Atsuta, N., Nakamura, R., Akiyama, T., Hadano, S., Aoki, M., Saya, H., Sobue, G., and Okano, H. (2018). Modeling sporadic ALS in iPSC-derived motor neurons identifies a potential therapeutic agent. Nat. Med. *24*, 1579–1589. https://doi.org/10.1038/s41591-018-0140-5.

Fujimori, K., Matsumoto, T., Kisa, F., Hattori, N., Okano, H., and Akamatsu, W. (2017). Escape from Pluripotency via Inhibition of TGF-β/BMP and Activation of Wnt Signaling Accelerates Differentiation and Aging in hPSC Progeny Cells. Stem Cell Rep. *9*, 1675–1691. https://doi.org/10.1016/j.stemcr.2017.09.024.

Goto, K., Imamura, K., Komatsu, K., Mitani, K., Aiba, K., Nakatsuji, N., Inoue, M., Kawata, A., Yamashita, H., Takahashi, R., and Inoue, H. (2017). Simple derivation of spinal motor neurons from ESCs/iPSCs using sendai virus vectors. Mol. Ther. Methods Clin. Dev. *4*, 115–125. https://doi.org/10.1016/j.omtm.2016.12.007.

Guo, W., Naujock, M., Fumagalli, L., Vandoorne, T., Baatsen, P., Boon, R., Ordovás, L., Patel, A., Welters, M., Vanwelden, T., et al. (2017). HDAC6 inhibition reverses axonal transport defects in motor neurons derived from FUS-ALS patients. Nat. Commun. *8*, 861. https://doi.org/10.1038/s41467-017-00911-y.

Imaizumi, Y., Okada, Y., Akamatsu, W., Koike, M., Kuzumaki, N., Hayakawa, H., Nihira, T., Kobayashi, T., Ohyama, M., Sato, S., et al. (2012). Mitochondrial dysfunction associated with increased oxidative stress and α-synuclein accumulation in PARK2 iPSC-derived neurons and postmortem brain tissue. Mol. Brain *5*, 35. https://doi.org/10.1186/1756-6606-5-35.

Imamura, K., Izumi, Y., Watanabe, A., Tsukita, K., Woltjen, K., Yamamoto, T., Hotta, A., Kondo, T., Kitaoka, S., Ohta, A., et al. (2017). The Src/c-Abl pathway is a potential therapeutic target in amyotrophic lateral sclerosis. Sci. Transl. Med. *9*, eaaf3962. https://doi.org/10.1126/scitranslmed.aaf3962.

Ito, D., Morimoto, S., Takahashi, S., Okada, K., Nakahara, J., and Okano, H. (2023). Maiden voyage: induced pluripotent stem cell-based drug screening for amyotrophic lateral sclerosis. Brain *146*, 13–19. https://doi.org/10.1093/brain/awac306.

Kim, D., Paggi, J.M., Park, C., Bennett, C., and Salzberg, S.L. (2019). Graph-based genome alignment and genotyping with HISAT2 and HISAT-genotype. Nat. Biotechnol. *37*, 907–915. https://doi.org/10.1038/s41587-019-0201-4.

Kwiatkowski, T.J., Jr., Bosco, D.A., LeClerc, A.L., Tamrazian, E., Vanderburg, C.R., Russ, C., Davis, A., Gilchrist, J., Kasarskis, E.J., Munsat, T., et al. (2009). Mutations in the FUS/TLS Gene on Chromosome 16 Cause Familial Amyotrophic Lateral Sclerosis. Science *323*, 1205–1208. https://doi.org/10.1126/science.1166066.

Lagier-Tourenne, C., and Cleveland, D.W. (2009). Rethinking ALS: The FUS about TDP-43. Cell *136*, 1001–1004. https://doi.org/10.1016/j.cell.2009.03.006.

Lamas, N.J., and Roybon, L. (2021). Harnessing the Potential of Human Pluripotent Stem Cell-Derived Motor Neurons for Drug Discovery in Amyotrophic Lateral Sclerosis: From the Clinic to the Laboratory and Back to the Patient. Front. Drug. Discov. *1*, 773424. https://doi.org/10.3389/fddsv.2021.773424.

Leventoux, N., Morimoto, S., Hara, K., Nakamura, S., Ozawa, F., Mitsuzawa, S., Akiyama, T., Nishiyama, A., Suzuki, N., Warita, H., et al. (2020). Generation of an ALS human iPSC line KEIOi001-A from peripheral blood of a Charcot disease-affected patient carrying TARDBP p.N345K heterozygous SNP mutation. Stem Cell Res. *47*, 101896. https://doi.org/10.1016/j.scr.2020.101896.

Lituma, P.J., Woo, E., O'hara, B.F., Castillo, P.E., Sibinga, N.E.S., and Nandi, S. (2021). Altered synaptic connectivity and brain function

in mice lacking microglial adapter protein Iba1. Proc. Natl. Acad. Sci. USA *118*, e2115539118. https://doi.org/10.1073/pnas.2115539118.

Love, M.I., Huber, W., and Anders, S. (2014). Moderated estimation of fold change and dispersion for RNA-seq data with DESeq2. Genome Biol. *15*, 550. https://doi.org/10.1186/s13059-014-0550-8.

Mattiazzi Usaj, M., Yeung, C.H.L., Friesen, H., Boone, C., and Andrews, B.J. (2021). Single-cell image analysis to explore cell-to-cell heterogeneity in isogenic populations. Cell Syst. *12*, 608–621. https://doi.org/10.1016/j.cels.2021.05.010.

Morimoto, S., Takahashi, S., Ito, D., Daté, Y., Okada, K., Kato, C., Nakamura, S., Ozawa, F., Chyi, C.M., Nishiyama, A., et al. (2023). Phase 1/2a clinical trial in ALS with ropinirole, a drug candidate identified by iPSC drug discovery. Cell Stem Cell *30*, 766–780.e9. https://doi.org/10.1016/j.stem.2023.04.017.

Nakamura, R., Tohnai, G., Nakatochi, M., Atsuta, N., Watanabe, H., Ito, D., Katsuno, M., Hirakawa, A., Izumi, Y., Morita, M., et al. (2023). Genetic factors affecting survival in Japanese patients with sporadic amyotrophic lateral sclerosis: a genome-wide association study and verification in iPSC-derived motor neurons from patients. J. Neurol. Neurosurg. Psychiatry *94*, 816–824. https://doi.org/10.1136/jnnp-2022-330851.

Naujock, M., Stanslowsky, N., Bufler, S., Naumann, M., Reinhardt, P., Sterneckert, J., Kefalakes, E., Kassebaum, C., Bursch, F., Lojewski, X., et al. (2016). 4-Aminopyridine Induced Activity Rescues Hypoexcitable Motor Neurons from Amyotrophic Lateral Sclerosis Patient-Derived Induced Pluripotent Stem Cells. Stem Cell. *34*, 1563–1575. https://doi.org/10.1002/stem.2354.

Nguyen, T.B., Lac, Q., Abdi, L., Banerjee, D., Deng, Y., and Zhang, Y. (2022). Harshening stem cell research and precision medicine: The states of human pluripotent cells stem cell repository diversity, and racial and sex differences in transcriptomes. Front. Cell Dev. Biol. *10*, 1071243. https://doi.org/10.3389/fcell.2022.1071243.

Novelli, G., Spitalieri, P., Murdocca, M., Centanini, E., and Sangiuolo, F. (2022). Organoid factory: The recent role of the human induced pluripotent stem cells (hiPSCs) in precision medicine. Front. Cell Dev. Biol. *10*, 1059579. https://doi.org/10.3389/fcell.2022.1059579.

Okano, H., and Morimoto, S. (2022). iPSC-based disease modeling and drug discovery in cardinal neurodegenerative disorders. Cell Stem Cell *29*, 189–208. https://doi.org/10.1016/j.stem.2022.01.007.

Okano, H., Yasuda, D., Fujimori, K., Morimoto, S., and Takahashi, S. (2020). Ropinirole, a New ALS Drug Candidate Developed Using iPSCs. Trends Pharmacol. Sci. *41*, 99–109. https://doi.org/10.1016/j.tips.2019.12.002.

Okita, K., Matsumura, Y., Sato, Y., Okada, A., Morizane, A., Okamoto, S., Hong, H., Nakagawa, M., Tanabe, K., Tezuka, K.i., et al. (2011). A more efficient method to generate integration-free human iPS cells. Nat. Methods *8*, 409–412. https://doi.org/10.1038/nmeth.1591.

Palasantzas, V.E.J.M., Tamargo-Rubio, I., Le, K., Slager, J., Wijmenga, C., Jonkers, I.H., Kumar, V., Fu, J., and Withoff, S. (2023). iPSC-derived organ-on-a-chip models for personalized human genetics and pharmacogenomics studies. Trends Genet. *39*, 268–284. https://doi.org/10.1016/j.tig.2023.01.002.

Park, J., Kim, J., Lewy, T., Rice, C.M., Elemento, O., Rendeiro, A.F., and Mason, C.E. (2022). Spatial omics technologies at multimodal and single cell/subcellular level. Genome Biol. *23*, 256. https://doi.org/10.1186/s13059-022-02824-6.

Patro, R., Duggal, G., Love, M.I., Irizarry, R.A., and Kingsford, C. (2017). Salmon provides fast and bias-aware quantification of transcript expression. Nat. Methods *14*, 417–419. https://doi.org/10.1038/nmeth.4197.

Radivojevic, M., and Rostedt Punga, A. (2023). Functional imaging of conduction dynamics in cortical and spinal axons. Elife *12*, e86512. https://doi.org/10.7554/elife.86512.

Robinson, J.T., Thorvaldsdóttir, H., Thorvaldsdóttir, H., Guttman, M., Lander, E.S., Getz, G., and Mesirov, J.P. (2011). Integrative genomics viewer. Nat. Biotechnol. *29*, 24–26. https://doi.org/10.1038/nbt.1754.

Sagner, A., and Briscoe, J. (2019). Establishing neuronal diversity in the spinal cord: a time and a place. Development *146*, dev182154. https://doi.org/10.1242/dev.182154.

Sances, S., Bruijn, L.I., Chandran, S., Eggan, K., Ho, R., Klim, J.R., Livesey, M.R., Lowry, E., Macklis, J.D., Rushton, D., et al. (2016). Modeling ALS with motor neurons derived from human induced pluripotent stem cells. Nat. Neurosci. *19*, 542–553. https://doi.org/10.1038/nn.4273.

Santilli, G., Lamorte, G., Carlessi, L., Ferrari, D., Rota Nodari, L., Binda, E., Delia, D., Vescovi, A.L., and De Filippis, L. (2010). Mild Hypoxia Enhances Proliferation and Multipotency of Human Neural Stem Cells. PLoS One *5*, e8575. https://doi.org/10.1371/journal.pone.0008575.

Schenke, M., Prause, H.C., Bergforth, W., Przykopanski, A., Rummel, A., Klawonn, F., and Seeger, B. (2021). Human-Relevant Sensitivity of iPSC-Derived Human Motor Neurons to BoNT/A1 and B1. Toxins *13*, 585. https://doi.org/10.3390/toxins13080585.

Sepehrimanesh, M., and Ding, B. (2020). Generation and optimization of highly pure motor neurons from human induced pluripotent stem cells via lentiviral delivery of transcription factors. Am. J. Physiol. Cell Physiol. *319*, C771–C780. https://doi.org/10.1152/ajpcell.00279.2020.

Shin, H.Y., Pfaff, K.L., Davidow, L.S., Sun, C., Uozumi, T., Yanagawa, F., Yamazaki, Y., Kiyota, Y., and Rubin, L.L. (2018). Using Automated Live Cell Imaging to Reveal Early Changes during Human Motor Neuron Degeneration. eNeuro *5*. ENEURO.0001–18.2018. https://doi.org/10.1523/ENEURO.0001-18.2018.

Sreedharan, J., Blair, I.P., Tripathi, V.B., Hu, X., Vance, C., Rogelj, B., Ackerley, S., Durnall, J.C., Williams, K.L., Buratti, E., et al. (2008). TDP-43 Mutations in Familial and Sporadic Amyotrophic Lateral Sclerosis. Science *319*, 1668–1672. https://doi.org/10.1126/science.1154584.

Stifani, N., Thompson, L., and Institute, N.; Australia (2014). Motor neurons and the generation of spinal motor neuron diversity. Front. Cell. Neurosci. *8*, 293. https://doi.org/10.3389/fncel.2014.00293.

Takahashi, K., Tanabe, K., Ohnuki, M., Narita, M., Ichisaka, T., Tomoda, K., and Yamanaka, S. (2007). Induction of Pluripotent Stem

Cells from Adult Human Fibroblasts by Defined Factors. Cell *131*, 861–872. https://doi.org/10.1016/j.cell.2007.11.019.

Takahashi, K., and Yamanaka, S. (2006). Induction of Pluripotent Stem Cells from Mouse Embryonic and Adult Fibroblast Cultures by Defined Factors. Cell *126*, 663–676. https://doi.org/10.1016/j.cell.2006.07.024.

Takeda, T., Iijima, M., Shimizu, Y., Yoshizawa, H., Miyashiro, M., Onizuka, H., Yamamoto, T., Nishiyama, A., Suzuki, N., Aoki, M., et al. (2019). p.N345K mutation in TARDBP in a patient with familial amyotrophic lateral sclerosis: An autopsy case. Neuropathology *39*, 286–293. https://doi.org/10.1111/neup.12559.

Tamaoka, A., Arai, M., Itokawa, M., Arai, T., Hasegawa, M., Tsuchiya, K., Takuma, H., Tsuji, H., Ishii, A., Watanabe, M., et al. (2010). TDP-43 M337V Mutation in Familial Amyotrophic Lateral Sclerosis in Japan. Intern. Med. *49*, 331–334. https://doi.org/10.2169/internalmedicine.49.2915.

Thams, S., Lowry, E.R., Larraufie, M.H., Spiller, K.J., Li, H., Williams, D.J., Hoang, P., Jiang, E., Williams, L.A., Sandoe, J., et al. (2019). A Stem Cell-Based Screening Platform Identifies Compounds that Desensitize Motor Neurons to Endoplasmic Reticulum Stress. Mol. Ther. *27*, 87–101. https://doi.org/10.1016/j.ymthe.2018.10.010.

Vance, C., Rogelj, B., Hortobágyi, T., De Vos, K.J., Nishimura, A.L., Sreedharan, J., Hu, X., Smith, B., Ruddy, D., Wright, P., et al. (2009). Mutations in FUS, an RNA Processing Protein, Cause Familial Amyotrophic Lateral Sclerosis Type 6. Science *323*, 1208–1211. https://doi.org/10.1126/science.1165942.

Wainger, B.J., Kiskinis, E., Mellin, C., Wiskow, O., Han, S.S.W., Sandoe, J., Perez, N.P., Williams, L.A., Lee, S., Boulting, G., et al. (2014). Intrinsic membrane hyperexcitability of amyotrophic lateral sclerosis patient-derived motor neurons. Cell Rep. *7*, 1–11. https://doi.org/10.1016/j.celrep.2014.03.019.

Watson, E.R., Taherian Fard, A., and Mar, J.C. (2021). Computational Methods for Single-Cell Imaging and Omics Data Integration. Front. Mol. Biosci. *8*, 768106. https://doi.org/10.3389/fmolb.2021.768106.

Wetts, R., and Vaughn, J.E. (1994). Choline acetyltransferase and NADPH diaphorase are co-expressed in rat spinal cord neurons. Neuroscience *63*, 1117–1124. https://doi.org/10.1016/0306-4522(94)90577-0.

Xu, H.J., Yao, Y., Yao, F., Chen, J., Li, M., Yang, X., Li, S., Lu, F., Hu, P., He, S., et al. (2023). Generation of functional posterior spinal motor neurons from hPSCs-derived human spinal cord neural progenitor cells. Cell Regen. *12*, 15. https://doi.org/10.1186/s13619-023-00159-6.

Stem Cell Reports, Volume *20*

Supplemental Information

Swift induction of human spinal lower motor neurons and robust ALS cell screening via single-cell imaging

Selena Setsu, Satoru Morimoto, Shiho Nakamura, Fumiko Ozawa, Kagistia Hana Utami, Ayumi Nishiyama, Naoki Suzuki, Masashi Aoki, Yukio Takeshita, Yukihide Tomari, and Hideyuki Okano

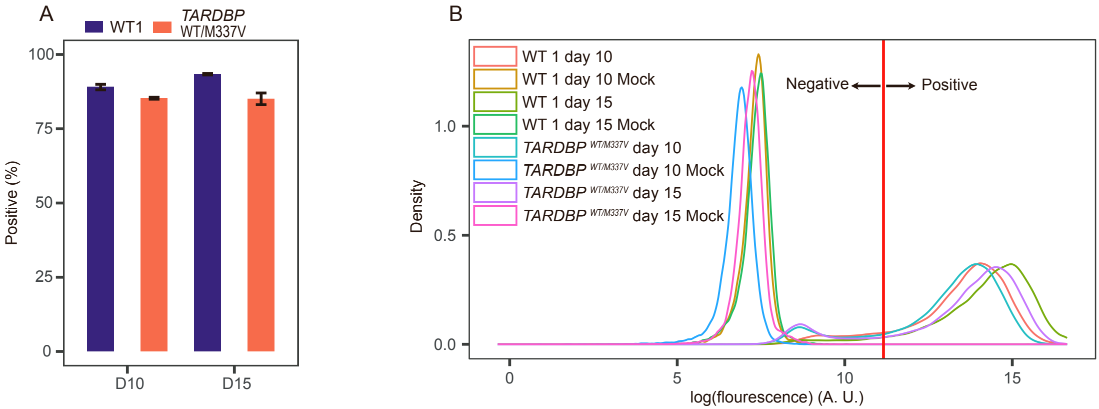

Figure S1 High induction efficiency was confirmed by FACS analysis using Hb9 promoter::mGreenLantern lentivirus

(A) Result of three technical replicates. (B) Gating strategy for negative and positive cells. For mock condition, n = 1. For the other conditions three replicates were combined in this figure.

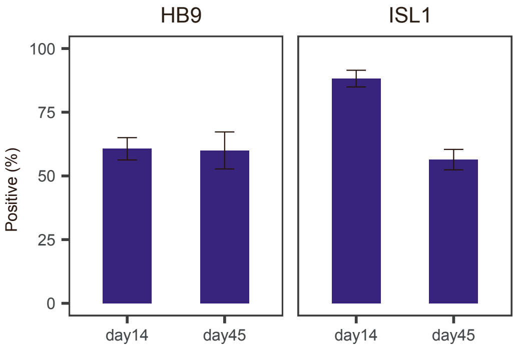

Figure S2 Differentiation efficiency at day 14 and day 45

The error bars are standard deviation. n = 8 biological replicates (different cell lines).

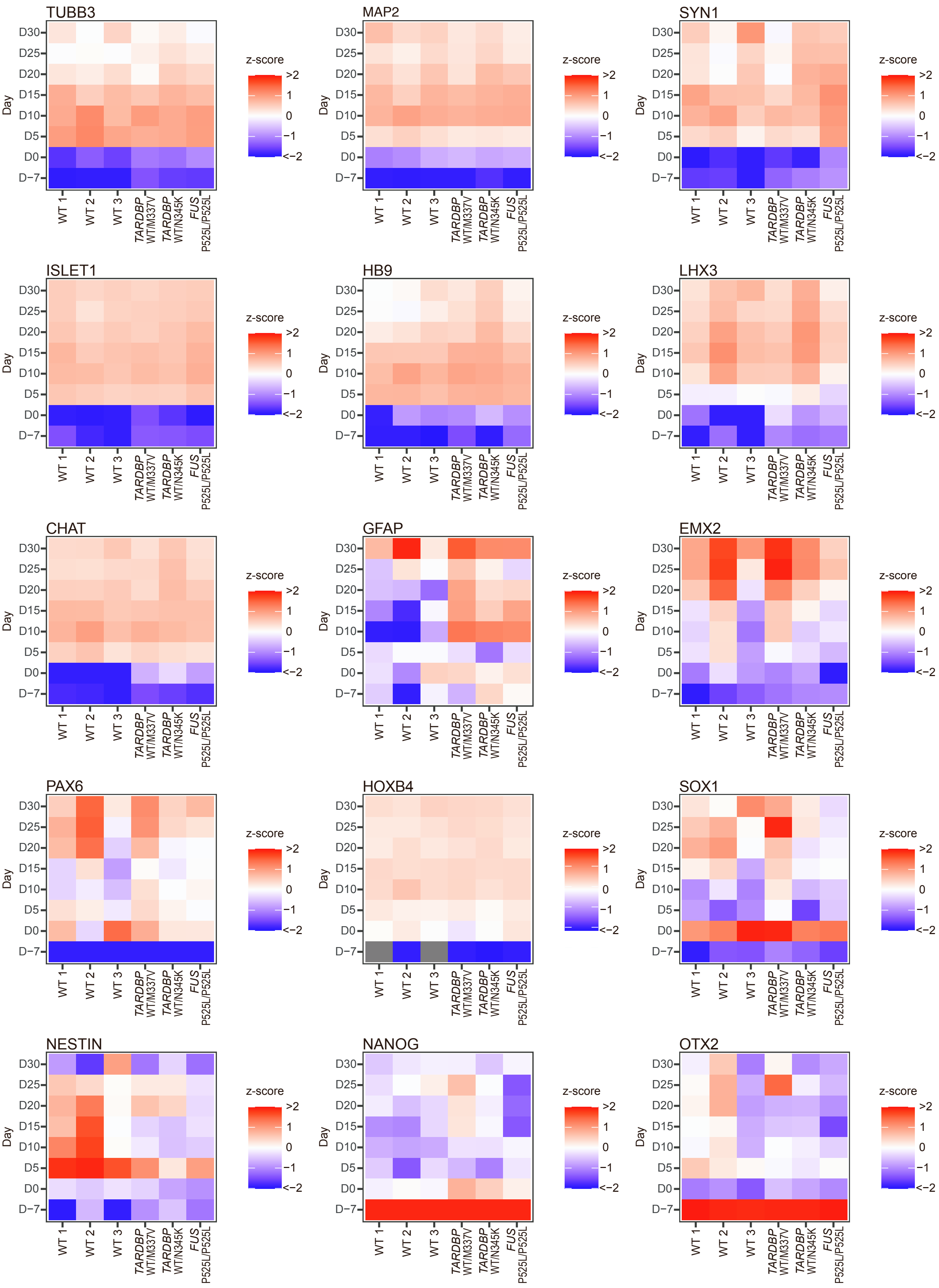

Figure S3 qPCR confirmed LMN characteristic expression until day 30

The expression of each gene is normalized using its z-score. n = 3 technical replicates. Grey represents missing data.

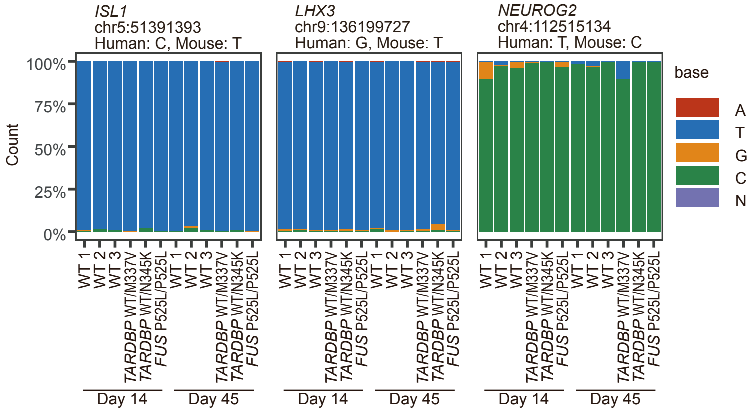

Figure S4 ISL1, LHX3 and NEUROG2 are mostly exogenously expressed in the induced LMNs.

Composition of bases at a location where the sequence of mouse and human are different.

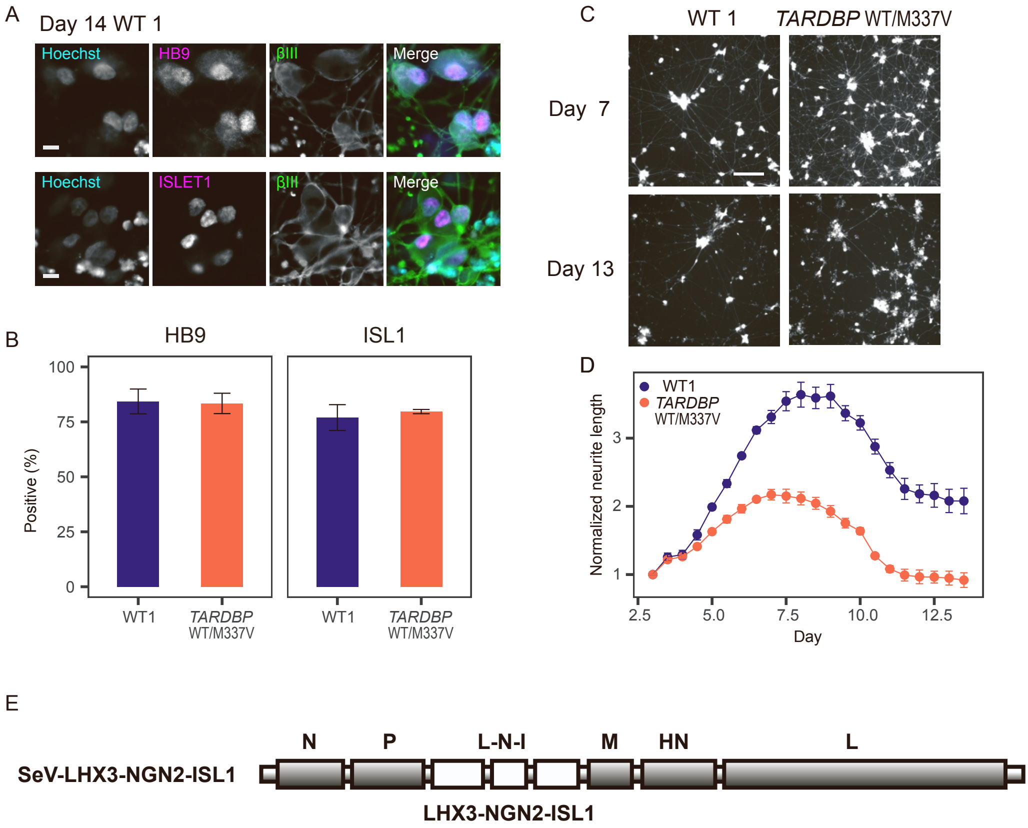

Figure S5 LMN induction using human transcription factors

(A) Representative images. (B) differentiation efficiency at day 14. The error bars are the standard deviation. n = 3 technical replicates. (C) mGreenLantern images of LMNs. (D) Total neurite length of each well was normalized by dividing by corresponding neurite length at day 3. n = 3 technical replicates. (E) Structure of the Sendai virus vector: gray boxes indicate Sendai virus genes, while white boxes represent inserted transcription factors.

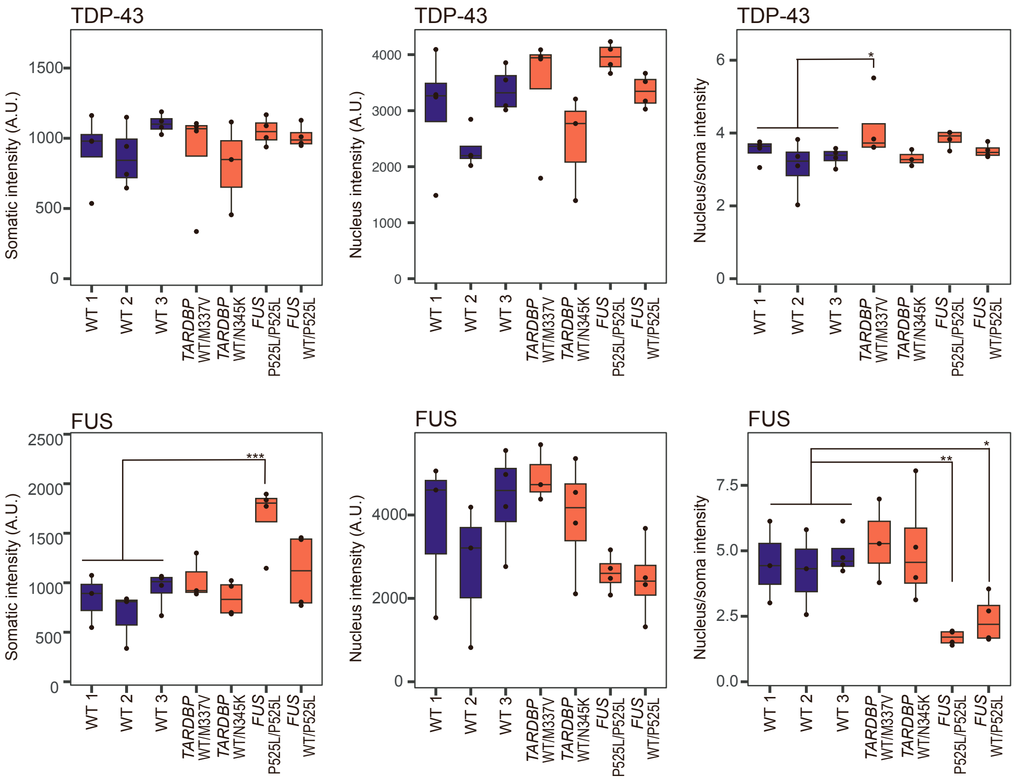

Figure S6 Depletion of FUS proteins from the nucleus in FUS mutant LMNs.

Left panel: Mean intensity of the somatic segment in each cell. Middle panel: Mean intensity of the nucleus segment in each cell. Right panel: Ratio of the mean intensity in the nucleus to mean intensity in the somatic segment of each cell. Results from three to four independent experiments were obtained, and a two-tailed Dunnett's test was performed. All tests were conducted against control cell lines. Unlabeled differences are not significant. *$p < 0.05$, **$p < 0.01$; ***$p < 0.001$.

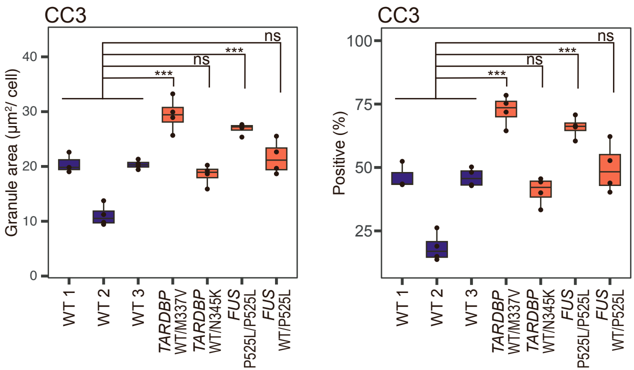

Figure S7 CC3 staining shows increased signal in ALS cell lines

Results from three to four independent experiments were obtained, and a two-tailed Dunnett's test was performed. *$p < 0.05$; **$p < 0.01$; ***$p < 0.001$; ns, non-significant.

Table S1

Number of Analyzed Neurons	23

Axon Metrics	Mean
Neuron Conduction Velocity [m/s]	0.539
Total Axon Length [μm]	2087.665
Longest Branch Length [μm]	864.265
Longest distance from Initiation Site [μm]	920.725
Longest Latency [ms]	2.668
Amplitude at Initiation Site [μV]	116.93

Table S2

Metric	Mean	P90
Firing Rate [Hz]	1.06	2.56
Spike Amplitude [µV]	35.97	52.34
Inter-Spike Interval [ms]	91.73	150.68

Table S3

Name	In the paper	Genotype	Age	Sex	Race	RIKEN BRC ID	Reprogramming methodology	Passage number	Reference
201B7	WT 1	wild type	36	female	Caucasian	HPS0063	Fibroblast, Retrovirus	< 35	Takahashi et al., 2007
1210B2	-	wild type	29	female	Caucasian	HPS4293	Peripheral Blood Mononuclear Cells, Episomal	< 35	Sugai et al., 2016
WD39	WT 2	wild type	16	female	Japanese	NA	Fibroblast, Retrovirus	< 35	Imaizumi, Y. et al.
414C2	WT 3	wild type	36	female	Caucasian	HPS4292	Fibroblast, Episomal	< 35	Okita et al., 2011
A3411	TARDBP WT/M337V	*TARDBP* WT/M337V	62	female	Japanese	HPS0292	Fibroblast, Episomal	< 35	Egawa et al., 2012
SM4-4-5	TARDBP WT/N345K	*TARDBP* WT/N345K*	63	male	Japanese	NA	T cells, Episomal	< 35	Leventoux et al., 2020
FUS-008-1-E6	FUS P525L/P525L	*FUS* P525L/P525L (edited from 201B7)	36	female	Caucasian	NA	NA	< 35	This paper
FUS-008-1-G2	FUS WT/P525L	*FUS* WT/P525L (edited from 201B7)	36	female	Caucasian	NA	NA	< 35	This paper
SM30 No.4	SOD1 ALS	*SOD1* WT/L39R	45	male	Japanese	NA	T cells, Episomal	< 35	This paper
30KALS-11	sporadic ALS	NA	70	male	Japanese	NA	T cells, Episomal	< 35	Morimoto et al., 2023
C9orf72 148-1 No.3	C9orf72 ALS 1	*C90rf72* repeat expansion	63	male	Japanese	NA	T cells, Episomal	< 35	Li et al., 2023
C9orf72 148-4 No.2	C9orf72 ALS 2	*C90rf72* repeat expansion	63	female	Japanese	NA	T cells, Episomal	< 35	This paper

*Medical conditon of SM4-4-5

**Family history**	three cousins with ALS
**Onset of disease**	at the age of 61
**First symptom**	clumsiness in the right hand
**Dementia**	No
**Death**	at the age of 64 due to pneumonia

*Medical conditon of C9orf72 148-1

**Family history**	one sister and one aunt with ALS
**Onset of disease**	at the age of 58
**First symptom**	muscle weakness in the left leg
**Dementia**	No

*Medical conditon of C9orf72 148-4

**Family history**	one brother and one aunt with ALS
**Onset of disease**	at the age of 57
**First symptom**	muscle weakness in the right leg
**Dementia**	No

Table S4

Target	Forward	Reverse
ACTB	TGAAGTGTGACGTGGACATC	GGAGGAGCAATGATCTTGAT
HB9	GTCCACCGCGGGCATGATCC	TCTTCACCTGGGTCTCGGTGAGC
ISLET1	AGCAGCCCAATGACAAAACT	CTGAAAAATTGACCAGTTGCTG
CHAT	GGAGGCGTGGAGCTCAGCGACACC	CGGGGAGCTCGCTGACGGAGTCTG
TUBB3	ATTTCATCTTTGGTCAGAGTGGGGC	TGCAGGCAGTCGCAGTTTTCAC
MAP2	GGCCCAAGCTAAAGTTGGTTCTC	GCAGTGACATCCTCAGCCAAAG
Nanog	TGAACCTCAGCTACAAACAG	TGGTGGTAGGAAGAGTAAAG
Nestin	TTCCCTCAGCTTTCAGGACCCCAA	AAGGCTGGCACAGGTGTCTCAA
GFAP	TGTGAGGCAGAAGCTCCAGGATGA	AGGGTGGCTTCATCTGCTTCCTGT
HOXB4	ACGTGAGCACGGTAAACCCCAA	ATTCCTTCTCCAGCTCCAAGACCT
LHX3	TGCAGGTTTGGTTCCAGAACCGCC	GCCAGGCCTCCATGCTCCAGGGAG
OTX2	ACAAGTGGCCAA TTCACTCC	GAGGTGGACAAGGGA TCTGA
EMX2	GCTTCTAAGGCTGGAACACG	CCAGCTTCTGCCTTTTGAAC
PAX6	ACCACACCGGTTTCCTCCTTCACA	TTGCCATGGTGAAGCTGGGCAT
SOX1	GATCAGCAAGCGCCTGGGGG	AGCAGCGTCTTGGTCTTGCGG
SYN1	ACTTCTGTGACAAGCCCTGG	ACCACGGGGTACGTTGTACT

Table S5

Name	Item number	Host	Isotype	Firm	Dilution
TDP-43 Polyclonal antibody	10782-2-AP	Rabbit	IgG	Proteintech	1:500
FUS Polyclonal Antibody	A300-293A	Rabbit	IgG	Bethyl Laboratories	1:1000
MNR2/HB9/Mnx1 Monoclonal antibody	81.5C10-C	Mouse	IgG1	DSHB	1:150
anti-isl1	39.4D5-C	Mouse	IgG2b	DSHB	1:1000
Purified anti-Tubulin β 3 (TUBB3) Antibody	801202	Mouse	IgG2a	BioLegend	1:2000
Cleaved Caspase-3 (Asp175) Antibody	9661	Rabbit	IgG	Cell Signaling	1:500
Goat anti-Rabbit IgG (H+L) Highly Cross-Adsorbed Secondary Antibody, Alexa Fluor™ 555	A21429	Goat	anti Rabbit IgG	Invitrogen	1:2000
Goat anti-Mouse IgG1 Cross-Adsorbed Secondary Antibody, Alexa Fluor™ 488	A21121	Goat	anti Mouse IgG1	Invitrogen	1:2000
Goat anti-Mouse IgG2a Cross-Adsorbed Secondary Antibody, Alexa Fluor™ 647	A21241	Goat	anti Mouse IgG2a	Invitrogen	1:2000
Goat anti-Mouse IgG2b Cross-Adsorbed Secondary Antibody, Alexa Fluor™ 488	A21141	Goat	anti Mouse IgG2b	Invitrogen	1:2000

Table S6

cell line	target	Class Name	cell count
201B7	HB9	Live_Negative	6665
201B7	HB9	Live_Positive	11269
1210B2	HB9	Live_Negative	5465
1210B2	HB9	Live_Positive	8569
WD39	HB9	Live_Negative	3920
WD39	HB9	Live_Positive	16623
414C2	HB9	Live_Negative	7141
414C2	HB9	Live_Positive	11936
A3411	HB9	Live_Negative	1989
A3411	HB9	Live_Positive	1657
FUS-E6	HB9	Live_Negative	3168
FUS-E6	HB9	Live_Positive	4947
FUS-G2	HB9	Live_Negative	1199
FUS-G2	HB9	Live_Positive	947
SM4	HB9	Live_Negative	10303
SM4	HB9	Live_Positive	4944
201B7	Islet1	Live_Negative	680
201B7	Islet1	Live_Positive	9567
1210B2	Islet1	Live_Negative	407
1210B2	Islet1	Live_Positive	5986
WD39	Islet1	Live_Negative	830
WD39	Islet1	Live_Positive	11300
414C2	Islet1	Live_Negative	783
414C2	Islet1	Live_Positive	10584
A3411	Islet1	Live_Negative	473
A3411	Islet1	Live_Positive	1306
FUS-E6	Islet1	Live_Negative	1373
FUS-E6	Islet1	Live_Positive	4122
FUS-G2	Islet1	Live_Negative	229
FUS-G2	Islet1	Live_Positive	1074
SM4	Islet1	Live_Negative	842
SM4	Islet1	Live_Positive	8726

Table S6, continued

cell line	target	Class Name	n	cell_count
201B7	FUS	Live_Positive	n1	258
201B7	FUS	Live_Positive	n2	414
201B7	FUS	Live_Positive	n3	285
WD39	FUS	Live_Positive	n1	561
WD39	FUS	Live_Positive	n2	718
WD39	FUS	Live_Positive	n3	1646
414C2	FUS	Live_Positive	n1	20
414C2	FUS	Live_Positive	n2	270
414C2	FUS	Live_Positive	n3	381
414C2	FUS	Live_Positive	n4	310
A3411	FUS	Live_Positive	n1	741
A3411	FUS	Live_Positive	n2	434
A3411	FUS	Live_Positive	n3	309
SM4	FUS	Live_Positive	n1	936
SM4	FUS	Live_Positive	n2	202
SM4	FUS	Live_Positive	n3	113
SM4	FUS	Live_Positive	n4	575
FUS-E6	FUS	Live_Positive	n1	34
FUS-E6	FUS	Live_Positive	n2	576
FUS-E6	FUS	Live_Positive	n3	106
FUS-E6	FUS	Live_Positive	n4	267
FUS-G2	FUS	Live_Positive	n1	86
FUS-G2	FUS	Live_Positive	n2	837
FUS-G2	FUS	Live_Positive	n3	709
FUS-G2	FUS	Live_Positive	n4	309
201B7	TDP	Live_Positive	n1	1312
201B7	TDP	Live_Positive	n2	489
201B7	TDP	Live_Positive	n3	1957
201B7	TDP	Live_Positive	n4	1362
WD39	TDP	Live_Positive	n1	1779
WD39	TDP	Live_Positive	n2	240
WD39	TDP	Live_Positive	n3	3571
WD39	TDP	Live_Positive	n4	2541
414C2	TDP	Live_Positive	n1	1176
414C2	TDP	Live_Positive	n2	1004
414C2	TDP	Live_Positive	n3	960
414C2	TDP	Live_Positive	n4	1572
A3411	TDP	Live_Positive	n1	507

Table S6, continued

A3411	TDP	Live_Positive	n2	870
A3411	TDP	Live_Positive	n3	651
A3411	TDP	Live_Positive	n4	415
SM4	TDP	Live_Positive	n1	1842
SM4	TDP	Live_Positive	n2	2728
SM4	TDP	Live_Positive	n3	424
FUS-E6	TDP	Live_Positive	n1	732
FUS-E6	TDP	Live_Positive	n2	467
FUS-E6	TDP	Live_Positive	n3	1016
FUS-E6	TDP	Live_Positive	n4	951
FUS-G2	TDP	Live_Positive	n1	1721
FUS-G2	TDP	Live_Positive	n2	977
FUS-G2	TDP	Live_Positive	n3	1256
FUS-G2	TDP	Live_Positive	n4	1015

Table S6, continued

cell line	target	day	Class Name	cell count
201B7	HB9	day14	Live_Negative	1110
201B7	HB9	day14	Live_Positive	340
201B7	HB9	day45	Live_Negative	3204
201B7	HB9	day45	Live_Positive	1353
1210B2	HB9	day14	Live_Negative	1319
1210B2	HB9	day14	Live_Positive	713
1210B2	HB9	day45	Live_Negative	3032
1210B2	HB9	day45	Live_Positive	1584
WD39	HB9	day14	Live_Negative	2258
WD39	HB9	day14	Live_Positive	1574
WD39	HB9	day45	Live_Negative	4070
WD39	HB9	day45	Live_Positive	2069
414C2	HB9	day14	Live_Negative	1926
414C2	HB9	day14	Live_Positive	924
414C2	HB9	day45	Live_Negative	1736
414C2	HB9	day45	Live_Positive	2727
A3411	HB9	day14	Live_Negative	773
A3411	HB9	day14	Live_Positive	738
A3411	HB9	day45	Live_Negative	4942
A3411	HB9	day45	Live_Positive	643
SM4	HB9	day14	Live_Negative	1394
SM4	HB9	day14	Live_Positive	543
SM4	HB9	day45	Live_Negative	5657
SM4	HB9	day45	Live_Positive	1206
FUS-E6	HB9	day14	Live_Negative	534
FUS-E6	HB9	day14	Live_Positive	384
FUS-E6	HB9	day45	Live_Negative	7507
FUS-E6	HB9	day45	Live_Positive	564
FUS-G2	HB9	day14	Live_Negative	1929
FUS-G2	HB9	day14	Live_Positive	535
FUS-G2	HB9	day45	Live_Negative	2507
FUS-G2	HB9	day45	Live_Positive	1736
201B7	ISL1	day14	Live_Negative	593
201B7	ISL1	day14	Live_Positive	2610
201B7	ISL1	day45	Live_Negative	2904
201B7	ISL1	day45	Live_Positive	721
1210B2	ISL1	day14	Live_Negative	315
1210B2	ISL1	day14	Live_Positive	1990

Table S6, continued

1210B2	ISL1	day45	Live_Negative	3599
1210B2	ISL1	day45	Live_Positive	1191
WD39	ISL1	day14	Live_Negative	755
WD39	ISL1	day14	Live_Positive	4601
WD39	ISL1	day45	Live_Negative	4317
WD39	ISL1	day45	Live_Positive	1377
414C2	ISL1	day14	Live_Negative	362
414C2	ISL1	day14	Live_Positive	3692
414C2	ISL1	day45	Live_Negative	2268
414C2	ISL1	day45	Live_Positive	1663
A3411	ISL1	day14	Live_Negative	511
A3411	ISL1	day14	Live_Positive	2707
A3411	ISL1	day45	Live_Negative	5626
A3411	ISL1	day45	Live_Positive	263
SM4	ISL1	day14	Live_Negative	242
SM4	ISL1	day14	Live_Positive	2619
SM4	ISL1	day45	Live_Negative	4434
SM4	ISL1	day45	Live_Positive	1077
FUS-E6	ISL1	day14	Live_Negative	285
FUS-E6	ISL1	day14	Live_Positive	1459
FUS-E6	ISL1	day45	Live_Negative	6170
FUS-E6	ISL1	day45	Live_Positive	411
FUS-G2	ISL1	day14	Live_Negative	629
FUS-G2	ISL1	day14	Live_Positive	3326
FUS-G2	ISL1	day45	Live_Negative	2238
FUS-G2	ISL1	day45	Live_Positive	950

Table S6, continued

cell line	target	Class Name	n	cell_count
201B7	CC3	Live_Positive	n1	1078
201B7	CC3	Live_Positive	n2	1506
201B7	CC3	Live_Positive	n3	789
WD39	CC3	Live_Positive	n1	2065
WD39	CC3	Live_Positive	n2	1435
WD39	CC3	Live_Positive	n3	3544
WD39	CC3	Live_Positive	n4	2974
414C2	CC3	Live_Positive	n1	1288
414C2	CC3	Live_Positive	n2	968
414C2	CC3	Live_Positive	n3	1030
414C2	CC3	Live_Positive	n4	1929
A3411	CC3	Live_Positive	n1	712
A3411	CC3	Live_Positive	n2	824
A3411	CC3	Live_Positive	n3	571
A3411	CC3	Live_Positive	n4	242
SM4	CC3	Live_Positive	n1	1765
SM4	CC3	Live_Positive	n2	928
SM4	CC3	Live_Positive	n3	2427
SM4	CC3	Live_Positive	n4	325
FUS-E6	CC3	Live_Positive	n1	718
FUS-E6	CC3	Live_Positive	n2	687
FUS-E6	CC3	Live_Positive	n3	1023
FUS-E6	CC3	Live_Positive	n4	599
FUS-G2	CC3	Live_Positive	n1	1598
FUS-G2	CC3	Live_Positive	n2	693
FUS-G2	CC3	Live_Positive	n3	1751
FUS-G2	CC3	Live_Positive	n4	994
